# A comprehensive study on 2D, 3D and solid tumor environment to explore a multifunctional biogenic nanoconjugate

**DOI:** 10.1038/s41598-021-87364-y

**Published:** 2021-04-22

**Authors:** B. S. Unnikrishnan, G. U. Preethi, T. T. Sreelekha

**Affiliations:** Laboratory of Biopharmaceuticals and Nanomedicine, Division of Cancer Research, Regional Cancer Centre (RCC), Medical College P.O., Thiruvananthapuram, Kerala 695011 India

**Keywords:** Biochemistry, Biological techniques, Cancer, Cell biology, Drug discovery, Oncology, Materials science

## Abstract

Emergence of nanotechnology created a drastic change in the field of cancer therapy due to their unique features in drug delivery and imaging. Polysaccharide based nanoparticles have received extensive attention in recent years as promising nanoparticle mediated drug delivery systems. Polysaccharides are endorsed with versatile merits including high drug encapsulation efficiency, efficient drug protection against chemical or enzymatic degradation, unique ability to create a controlled release and cellular internalization. In the current study, we have fabricated doxorubicin-loaded carboxymethylated PST001 coated iron oxide nanoparticles (DOX@CM-PST-IONPs) for better management of cancer. CM-PST coated iron oxide nanoparticles co-encapsulated with chemotherapeutic drug doxorubicin, can be utilized for targeted drug delivery. Biocompatible and non-toxic nanoconjugates was found to be effective in both 2-D and 3-D cell culture system with efficient cancer cell internalization. The bench-marked potential of CM-PIONPs to produce reactive oxygen species makes it a noticeable drug delivery system to compact neoplasia. These nanoconjugates can lay concrete on a better way for the elimination of cancer spheroids and tumor burden.

## Introduction

Superparamagnetic magnetite iron oxide nanoparticles (IONPs) have a ranging role in drug/ gene delivery system, separation and purification of biomolecule, hyperthermia therapy and contrast agent for bio imaging^1–3^. Ongoing research on IONPs revealed that the iron-dependent reactive oxygen species (ROS)-reliant cell death pathway was found to be prominent in lipid peroxidation mediated cell death^4,5^. A large array of polymers including natural and synthetic versions have been employed as coating agents for increasing the stability of IONPs; however, most of them tend to generate off-target effects in animal models^6^. PST001, a galactoxyloglucan isolated, purified and characterized from the seed kernel of *Tamarindus indica* exhibited excellent biocompatibility, biodegradability, solubility and thermostability^7–9^. PST001 is composed of 1, 4-linked-d-glucopyranose substituted at the O-6 position of glucopyranosyl residues with d-xylopyranose with some of the xylose residues are d-galactosylated at O-2, was capable with anticancer, antiangiogenic, and anti-metastatic activities^10–12^. Due to the presence of free reducing aldehyde groups, PST001 was employed for the biogenic fabrication of metallic NPs including gold, silver and copper NPs^13,14^. Owing to the outstanding physicochemical and biomedical features of PST001, we envisaged to use it as a template for the nanofabrication of magnetic nanoparticles.

An in-depth investigation on drug delivery systems revealed the use of many synthetic polymers which in turn cause undesirable side effects without credeble therapeutic output. Among the various drug-loaded iron oxide nanoparticles, carboxydextran coated IONPs catch attention for its high solubility and ease of synthesis^15,16^. However, most of the IONP based drug delivery systems tend to produce hypersensitive reactions among treated groups which makes it difficult to be used in the preclinical study^17,18^. Hence, we carboxylated PST001 to extend its capping efficiency on the metal surface and to load chemotherapeutic drugs for synergistic death kinetics mediated by ROS and drug with minimal side effects.

Recent progress in 3D cell culture models enabled researchers to replicate the tumor microarchitecture composed of cancer foci within a reactive stroma, which is populated by non-cancer cells such as fibroblasts and endothelial cells in supporting tumor growth and potential drug response^19,20^. Due to the excessive expenditure in the development and the generation of drug delivery systems along with undesired side effects and long-term health risks, nanoformulation for the treatment of malignant tumors has the lowermost grant in the drug development scenario. We are engrossed to find out whether the biocompatible DOX@CM-PIONPs are proficient to penetrate a 3-D glioblastoma model as well as in solid tumor models. A pH-responsive ROS generating tumor penetrating safe nano-drug delivery system could promote clinical translation in attenuating the tumor burden.

## Results and discussion

### Choice of materials

**Scheme 1:**
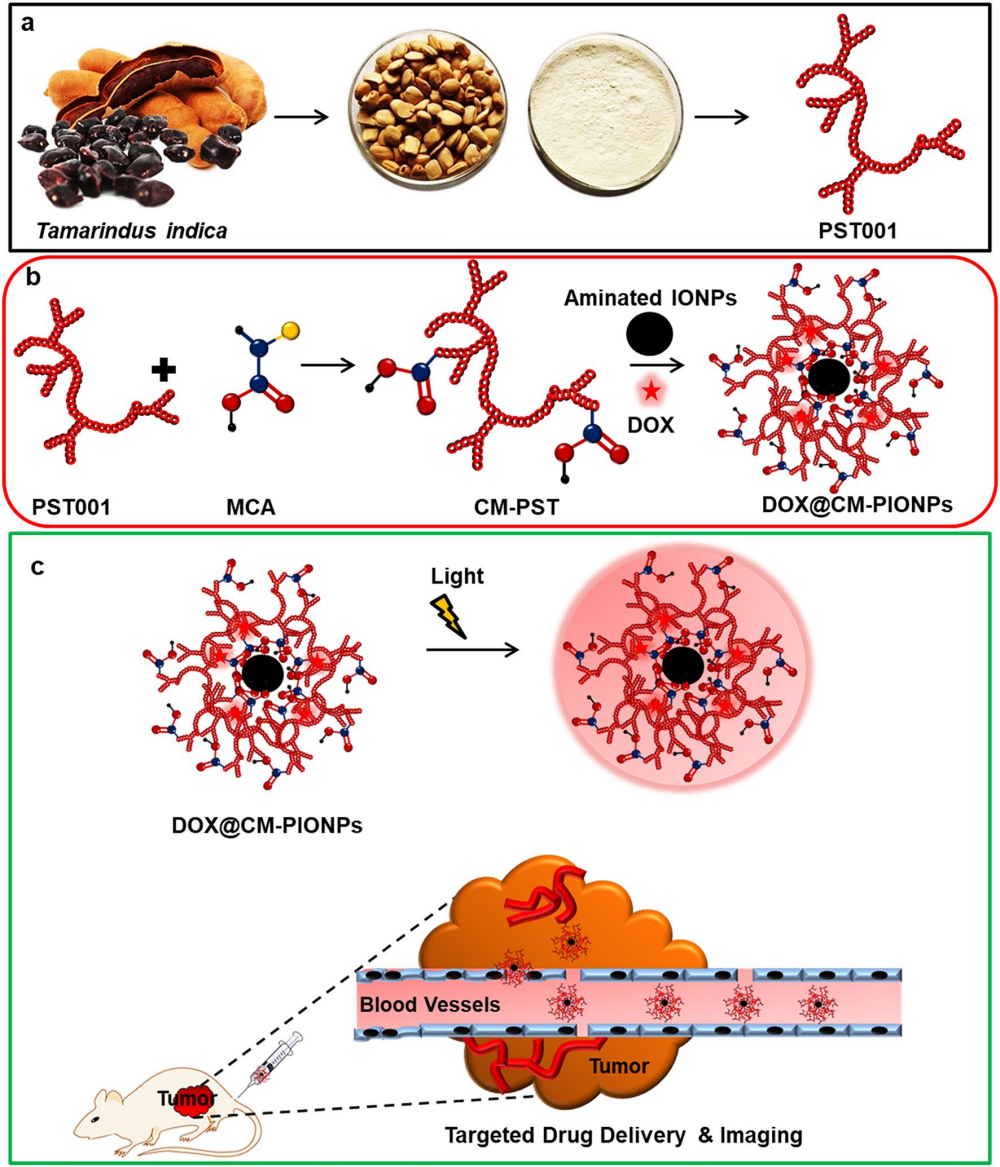
Overall scheme of the work. (**a**) Purification of the polysaccharide (PST001) from the seed kernel of Tamarindus indica (reprinted from https://www.etsy.com/in-en/listing/692397559/tamarind-tree-seed-tamarindus-indica and https://www.indiamart.com/proddetail/tamarind-kernel-powder-21080132233.html); (**b**) Steps involved in the production of biogenic DOX@CM-PIONPs. PST001 reacted with monochloroacetic acid (MCA) to generate carboxymethylated PST001 (CM-PST) which bounded the metal surface of aminated iron oxide nanoparticles (IONPs) and conjugated with doxorubicin (DOX) to generate DOX@CM-PIONPs; (**c**) Biomedical application of DOX@CM-PIONPs: In vivo administration of the particles favors its accumulation in tumor site which can be monitored from the fluorescence emission of the drug.

The polysaccharide PST001 isolated from the seed kernel of *Tamarindus indica* was used in the current study because of its appealing chemical, physical and biomedical properties. The ease of isolation and purification makes PST001 more affordable than any other polymers. Moreover, PST001 exhibited an appreciable level of tolerance in different temperatures and pHs^11^. Previous studies have reported biocompatibility with potent immunomodulating and antitumor properties of PST001 making it a promising drug carrier. PST001 based gold and doxorubicin (DOX) conjugated nanoparticles were found to be superior to the corresponding parent counterparts, suggesting the favourable role of a new cell-adaptive drug delivery nanosystem for the efficient treatment of cancer^21,22^. In view of the above-mentioned facts, the present study utilizes chemically synthesized anionic carboxymethylated PST001 (CM-PST) for the conjugation of aminated iron oxide nanoparticles and amino group bearing doxorubicin (DOX). An overview of the work is displayed in Scheme 1.

### Carboxymethylation of PST001

**Figure 1:**
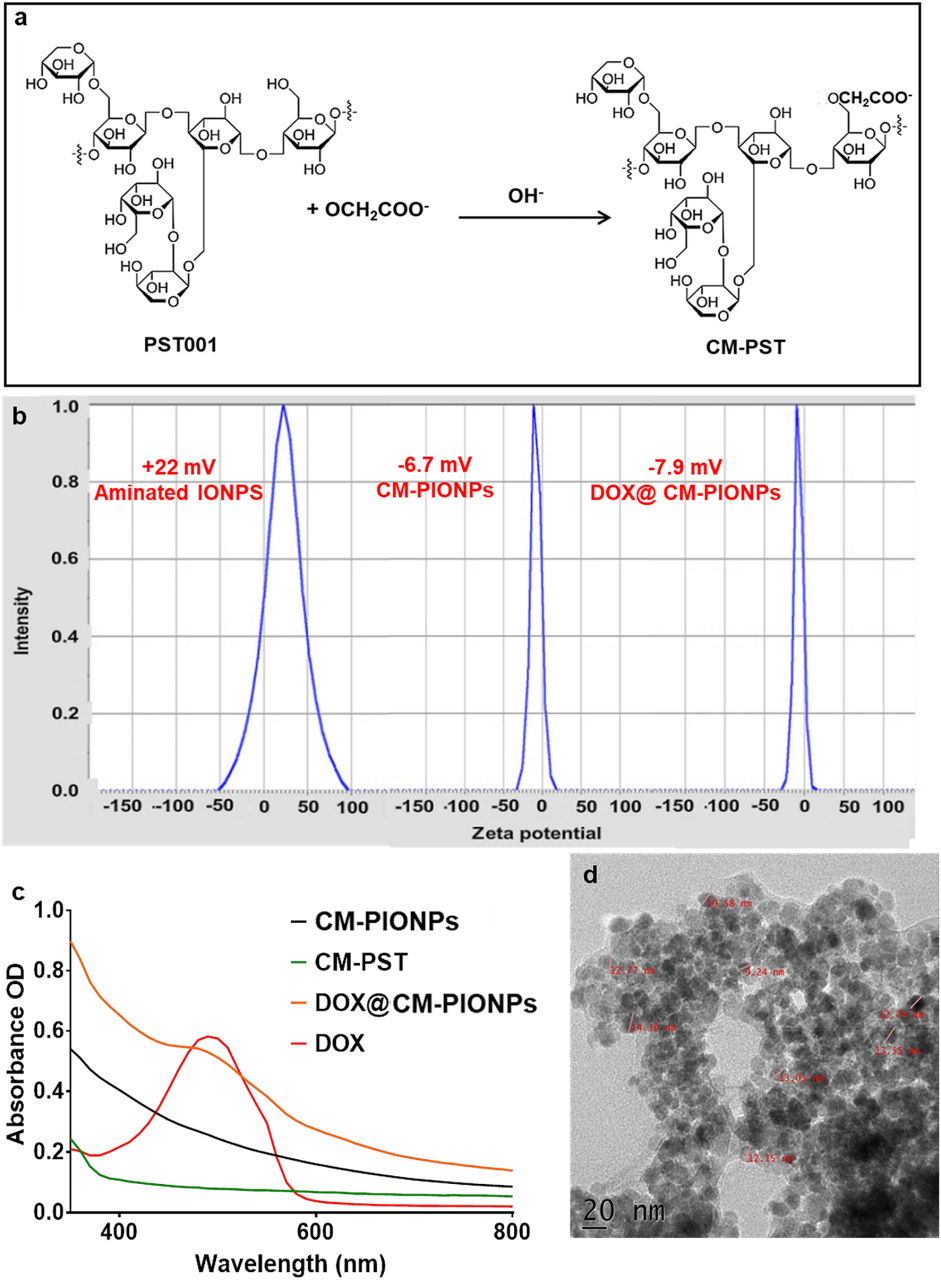
(**a**) Schematic representation of the formation of CM-PST in the presence of the alkali environment. (**b**) Zeta potential analysis. The decrease in the zeta potential of APTES coated iron oxide nanoparticles suggested the effective coating of CM-PST on the surface of the nanoparticles. (**c**) UV–Vis analysis. The data clearly shows the presence of doxorubicin in DOX@CM-PIONPs was with an absorbance maximum 490 nm; (**d**) HR-TEM image. DOX@CM-PIONPs found to be a spherical shape with a core size of 10 ± 5 nm.

Carboxymethylated PST001 conjugates were prepared using the previously reported method^23^; depending on various parameters used, in terms of alkali concentration and substituting agent as depicted in Fig. 1a. In alkaline pH, the hydroxyl group of PST001 will be activated and reacted with monochloroacetic acid (MCA) to yield CM-PST.

Fourier transform infrared spectroscopy (FTIR) analysis revealed that PST001 was carboxymethylated using monochloroacetic acid as shown in Fig S1. Structural analyses indicated that the presence of –COOH functional groups with sharp peaks at 1270 and 1213 cm^−1^. The physiological parameters of the modified PST001 were analyzed using sodium bicarbonate test and pH evaluation as indicated in Supplementary Table 1. It was found that pH was decreased to 5.77 in CM-PST compared to unmodified PST001 and also the production of carbon dioxide gas on reaction with NaHCO_3_ revealed the presence of acidic functional groups in modified CM-PST. The degree of substitution for the rate of conjugation was confirmed as described earlier^23^ illustrated a degree of substitution of 0.643.

### Fabrication of doxorubicin-loaded CM-PIONPs

CM-PST can be employed for capping iron oxide nanoparticles which can be further loaded with doxorubicin. Iron oxide nanoparticles (IONPs) were synthesized via chemical co-precipitation of Fe^2+^ and Fe^3+^ ions with an average diameter of 10 ± 2 nm (Fig S2). The silanization of IONPs was achieved using aminopropyltriethoxysilane (APTES) which will lead to the formation of Fe–O–Si bond between the nanoparticles and silane ligand^24^. The conjugation of APTES and IONP was confirmed by FTIR analysis. The presence of IONPs can be observed by a strong peak at 576 cm^−1^, especially for the Fe–O bond. Also, a peak at 1017 cm^−1^ and 1630 cm^−1^ corresponds to Si–O and amide bond respectively which indicated the presence of APTES coating. The sharp band at 3394 cm^−1^ can be referred to as the N–H stretching of the APTES coated IONPs. Due to the existence of APTES coating on the exterior surface of IONPs, a ζ potential value of + 22.9 was observed, but it has been changed to − 6.7 upon CM-PST coating on the surface of NPs (Fig. 1b). The decrease in the ζ potential of APTES coated IONPs suggested the effective coating of CM-PST on the surface of the nanoparticles. It was reported that superparamagnetic iron oxide nanoparticles with a carboxydextran shell resulted in higher cell uptake compared to nanoparticles with a dextran shell^25^. The FTIR spectrum of CM-PIONPs indicated the presence of –CONH bond at 1645 cm^−1^ and 3300 cm^−1^ corresponds to extensive hydrogen bonding between CM-PST and IONPs (Fig S3).

To find the importance of PST001 during the fabrication of DOX@CM-PIONPs, we have carried out various synthetic routes labelled ‘a’, ‘b, and ‘c’ (Fig S4). When route ‘a’ involved the synthesis of DOX loaded IONPs, route ‘b’ used the synthesis of DOX loaded aminated IONPs and route ‘c’ involved the preparation of DOX loaded CM-PIONPs. The particles synthesized via route ‘a’ and ‘b’ was precipitated and formed aggregates with huge hydrodynamic size. Only DOX@CM-PIONPs synthesized via route ‘c’ were stable with a low polydispersity index. The final supernatant of route ‘a’ and ‘b’ after magnetic separation exhibited the absorption maxima of doxorubicin while the supernatant of route ‘c’ showed less absorption spectrum of the drug. This indicated that the particles formed as a result of the first two methods were not successful while the particles by route ‘c’ were found to be stable drug-loaded magnetic nanoparticles (DOX@CM-PIONPs). The role of CM-PST001 in the stabilization of these nanoparticles was well understood from these data (Supplementary Table 2).

The CM-PIONPs were allowed to react with 1 mg/ml doxorubicin to yield an encapsulation efficiency of 870 µg per 10 mg nanoparticles. The presence of doxorubicin in the nanoparticles was confirmed by UV–Vis spectroscopy with a peak at 490 nm (Fig. 1c). HR-TEM analysis revealed spherically shaped particles with a core size of 10 ± 5 nm (Fig. 1d, Fig. S5). Due to the presence of polysaccharide, the hydrodynamic size of the particles was found to be relatively larger with a polydispersity index value of 0.594 (Figure S6). The DOX@CM-PST-IONPs have also shown a zeta potential of − 7.9 mV. It was reported that cationic nanoparticles caused genotoxicity and damage to vital organs like lungs/spleen while lesser negative nanoparticles appropriate to deliver the drug to the organs like the brain without triggering a systemic toxic effect^26^.

The release kinetics of doxorubicin from DOX@CM-PIONPs was evaluated under varying pHs at ambient temperature by HPLC analysis. It was found that doxorubicin displayed a sustained release for about 72 h in acidic pH (Figure S7). An acidic pH provides sufficient protons (H^+^) for the dissociation of amino-carboxyl interaction in DOX@CM-PIONPs that causes pH-dependent drug release behavior^27,28^. The nanosystem retards the drug release at neutral pH but promotes steady release at low pH. This is especially beneficial for drug delivery, where the acidic environment in the tumor site and late endosomes/lysosomes in cells facilities drug release from DOX@CM-PIONPs, while the discharge in blood and other normal tissues are slow decreasing undesirable side effects. It is worth mentioning that both CM-PIONPs and DOX@CM-PIONPs displayed appreciable stability for more than 120 days at room temperature (Supplementary Table 3).

### Evaluation of biocompatibility

**Figure 2:**
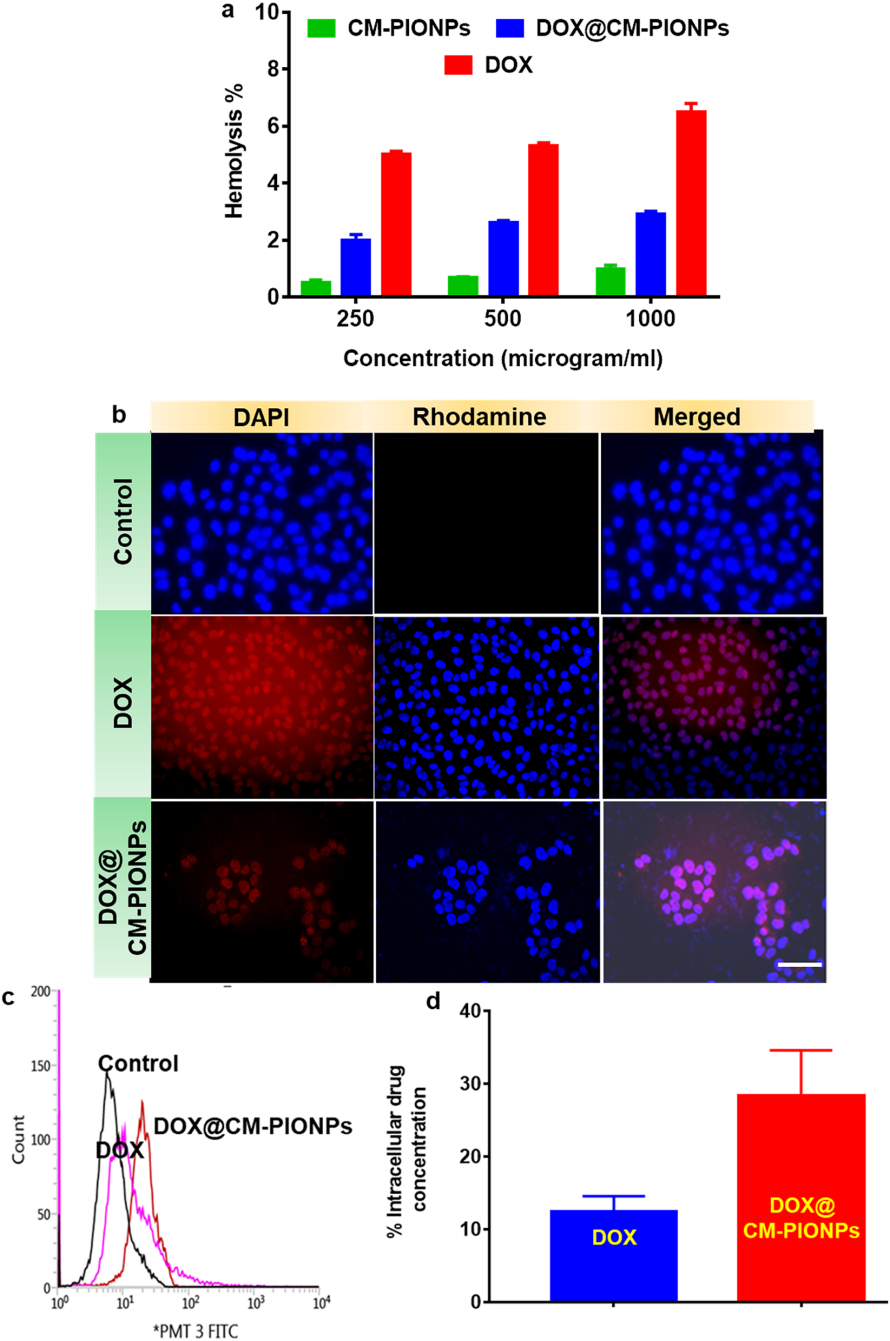
(**a**) Hemocompatibility assay. The result clearly shows that both CM-PIONPs and DOX@CM-PIONPs exhibited less than 3% hemolysis compared to DOX. (**b**) Cellular uptake of DOX and DOX@CM-PIONPs in A549 cells. DOX@CM-PIONPs was internalized within the cytosol of the cells after 3 h of incubation which proposes it’s as a drug delivery system. (**c**) Flow cytometric cellular uptake analysis. The cells treated with DOX@CM-PIONPs exhibited more fluorescence intensity (> 10^1^) compared to cells treated with DOX (**d**) Quantitative cellular uptake in A549 cells using HPLC. The uptake efficiency of nanoparticles in A549 was found to two-time more than the free drug. Scale bar corresponds to 50 µm. The experiments were carried out in triplicates and error bars denote standard deviations.

The biocompatibility of CM-PIONPs and DOX@CM-PIONPs was carried out to evaluate the suitability of the particles in the biological systems. To find the interaction of red blood cells (RBCs) with the nanoparticles, hemolysis assay was carried out with saline as negative control and distilled water as the positive control. Both naked and drug-loaded IONPs exhibited less than 3% hemolysis compared to the free doxorubicin (Fig. 2a). The phase-contrast microscopic image also revealed the absence of RBC aggregation in the presence of nanoparticles whereas the cells were found to be lysed in the positive control (Figure S8)^29,30^.

To confirm the non-toxic nature of the particles, cell-viability assays were performed against isolated peripheral human lymphocytes and IC-21 macrophages (Figure S9-10). Only 15% of cells were affected even at the highest concentration (1000 µg/ml) in the case of CM-PIONPs while the percent of non-viable cells on treatment with DOX@CM-PIONPs was reached up to 25% at its maximum concentration (100 µg/ml) (Figure S11). Many reports stated the toxic effects of IONPs even at lower concentrations but CM-PIONPs were appreciably safe^31,32^. Next, the anti-tumor effects were evaluated in human lung adenocarcinoma (A549), human glioblastoma (U-87MG) and murine melanoma (B16F10) cell lines by MTT (3-[4,5-dimethylthiazol-2yl]-2,5 diphenyltetrazolium) assay and live-dead staining. As showed in Figure S12, CM-PIONPs demonstrated no apparent cytotoxic effect in lower concentrations after incubation for 72 h in most of the cells except A549 and B16F10 at the highest concentration (1000 µg/ml). In both cell lines, the viability was reduced to 50% after incubation for 48 and 72 h, revealing that the magnetic nanoparticle can act as an anti-tumor agent at higher concentrations. Upon incubation with DOX@CM-PIONPs, most of the cells exhibited relatively higher toxicity than their parent drug molecule. The particles exhibited higher cytotoxicity compared with free doxorubicin at the same concentration after incubation for 72 h in A549 cells while it displayed a comparable anti-tumor effect in B16F10 melanoma cells. DOX@CM-PST-IONPs is suitable for a better therapeutic effect in a cancer cell-targeted manner. Similarly, U-87MG exhibited lesser cytotoxicity on treatment with NPs to the free drug (Supplementary Table 4). However, the therapeutic potential of nanoparticle can better be estimated in vivo tumor environment^33^.

Tumor cells presented nuclear condensation, fragmented nuclei and cell shrinkage after incubation with DOX@CM-PIONPs (Figure S13). To study the mechanism of CM-PIONPs on cell death, we have investigated the reactive oxygen species formation using 2ʹ,7ʹ-Dichlorofluorescin Diacetate (DCFDHA) assay in A549 and B16F10 cells. There was an increased level of ROS production in A549 cells compared to B16F10. The result was in agreement with the cell-viability analysis. Iron oxide particles cause oxidative stress in tumor cells causing the release of free radicals (O2^·–^ and HO^·^) via Fenton reactions to hinder the electron transport chain in mitochondria of live cells^34–36^. The synergistic effect of CM-PIONPs induced ROS production and drug-induced DNA damage leading to apoptosis in tumor cells with DOX@CM-PIONPs (Figure S14).

### Cellular internalization and mechanistic aspects

Further downstream experiments were designed with A549 cells. The uptake efficiency of particles was monitored upon comparison with free doxorubicin (Supplementary Table 5). DOX@CM-PIONPs were internalized within 3 h of incubation. Due to fluorescence emitted by doxorubicin, the particles were visible which is quantitatively analyzed from trypsinized cells (Fig. 2b). Compared to free drugs, the particles were accumulated inside the cell which is evident from flow cytometry. The side scatters (SSC) pattern in flow cytometry for nanoparticle treated cells exhibited the granulation in the cytosolic region due to the accumulation of the nanoparticles (Fig. 2c, Fig. S15)^37^. The uptake efficiency of nanoparticles in A549 cells was found to two-time more than the free drug as confirmed by HPLC (Fig. 2d).

**Figure 3:**
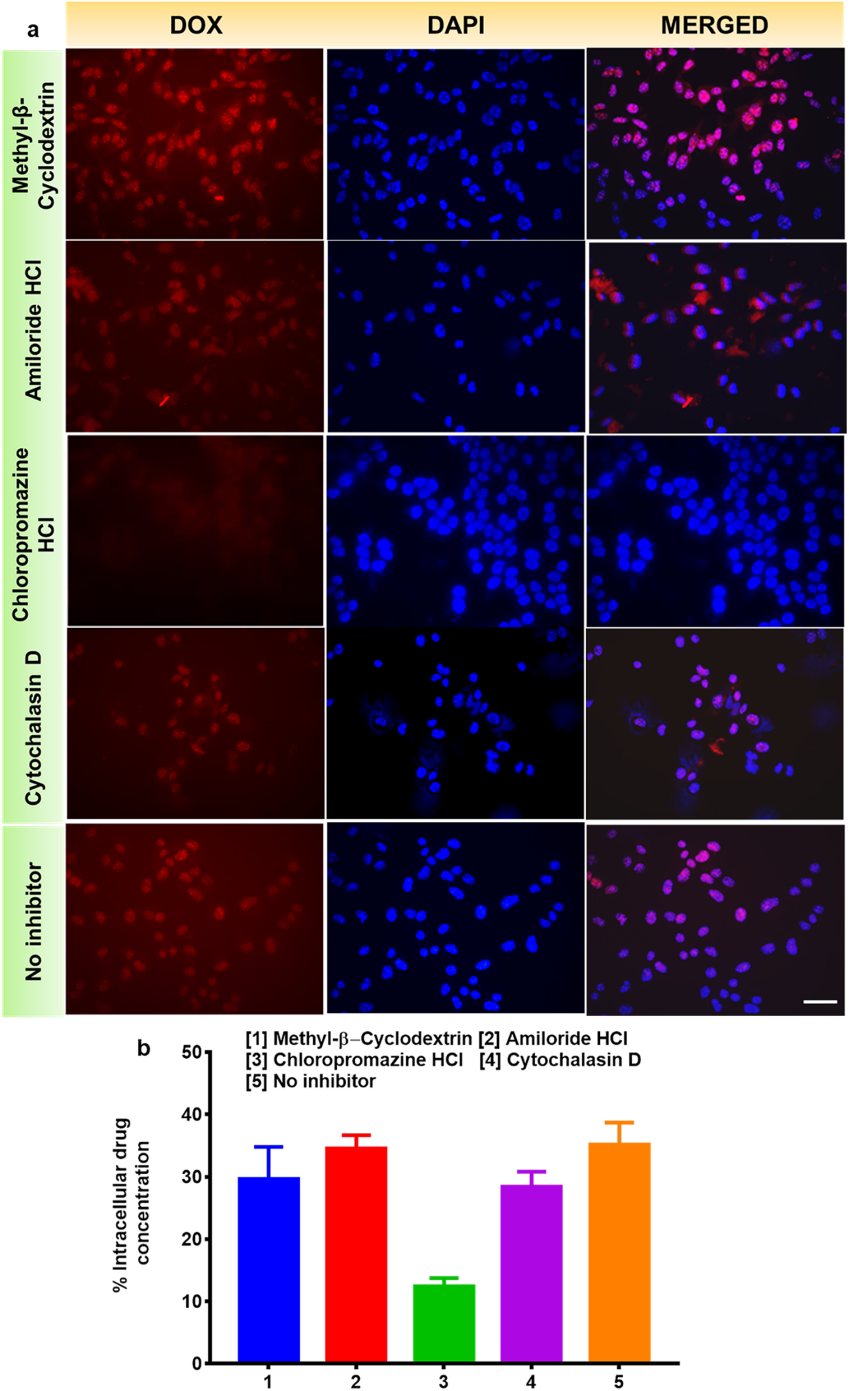
Confluent A549 cells were pre-treated with endocytotic inhibitors Methyl β cyclodextrin, Cytochalasin D, Chlorpromazine HCl and Amiloride HCl for 2 h, then treated with DOX@CM-PIONPs. (**a**) Fluorescent microscopic images of treated cells represent the DOX@CM-PIONPs were mediated by the clathrin-mediated endocytosis although combined effects of clathrin and macropinocytosis and (**b**) quantitative cellular uptake using HPLC suggested the clathrin-dependent endocytosis of nanoparticles. Scale bar corresponds to 50 µm. The experiments were carried out in triplicates and error bars denote standard deviations.

Next, we proceeded to the mechanistic aspects of cellular uptake of the particles. A549 cells treated with chlorpromazine HCl exhibited a significant decrease in the rate of intracellular drug uptake compared to other inhibitors suggesting the clathrin-dependent endocytosis of particles^38^. Dos Santos et al. established that transferrin is a ligand exclusively internalized via the clathrin-mediated endocytosis pathway, and thus its uptake is expected to be affected by treatment with chlorpromazine^39^. Chlorpromazine HCl inhibit clathrin-mediated endocytosis that inhibits the adapter proteins involved in the formation of clathrin pits^40^. The other three endocytosis inhibitors inhibited the caveolae-mediated endocytosis for Methyl β cyclodextrin, phagocytosis for Cytochalasin D and macropinocytosis for amiloride HCl were employed to determine the cellular uptake mechanisms of nanoparticles^41^. It could be concluded that the cellular uptake of DOX@CM-PIONPs was mainly mediated by the clathrin-mediated endocytosis. The mechanistic aspect of nanoparticle uptake suggested the clathrin-dependent endocytosis of nanoparticles (Fig. 3). Receptor-mediated endocytosis mediated by clathrin-coated pits in A549 cells treated with DOX@CM-PIONPs proposed the TRAIL-mediated uptake of nanoparticles that can further induce mitochondrial damage followed by cell death. This is in agreement with the previous studies from our laboratory stating the TRAIL-mediated uptake mechanism of PST001^11^.

### Therapeutic potential in a 3-D tumor glioblastoma model

Next, we have investigated the effect of these nanoparticles in the tumoroid models to create a mimicking scenario for the in vivo tumor microenvironment. The formation of spheroids was monitored after incubation of cells in agarose-coated 96-well plates for around 48 h. As anticipated, the concentration of seeded cells did not affect the size of the formed spheroid and the spheroid number increased proportionally to the seeding density (Figure S16). Glioblastoma spheroids (GB-spheroids) grew as homogenous cellular clumps and exhibited cell death during spheroid formation. Representative micrographs of spheroid formation as a function of time are indicated in Figure S17. Calcein AM staining of a representative GB-spheroid is shown in Figure S18.

**Figure 4:**
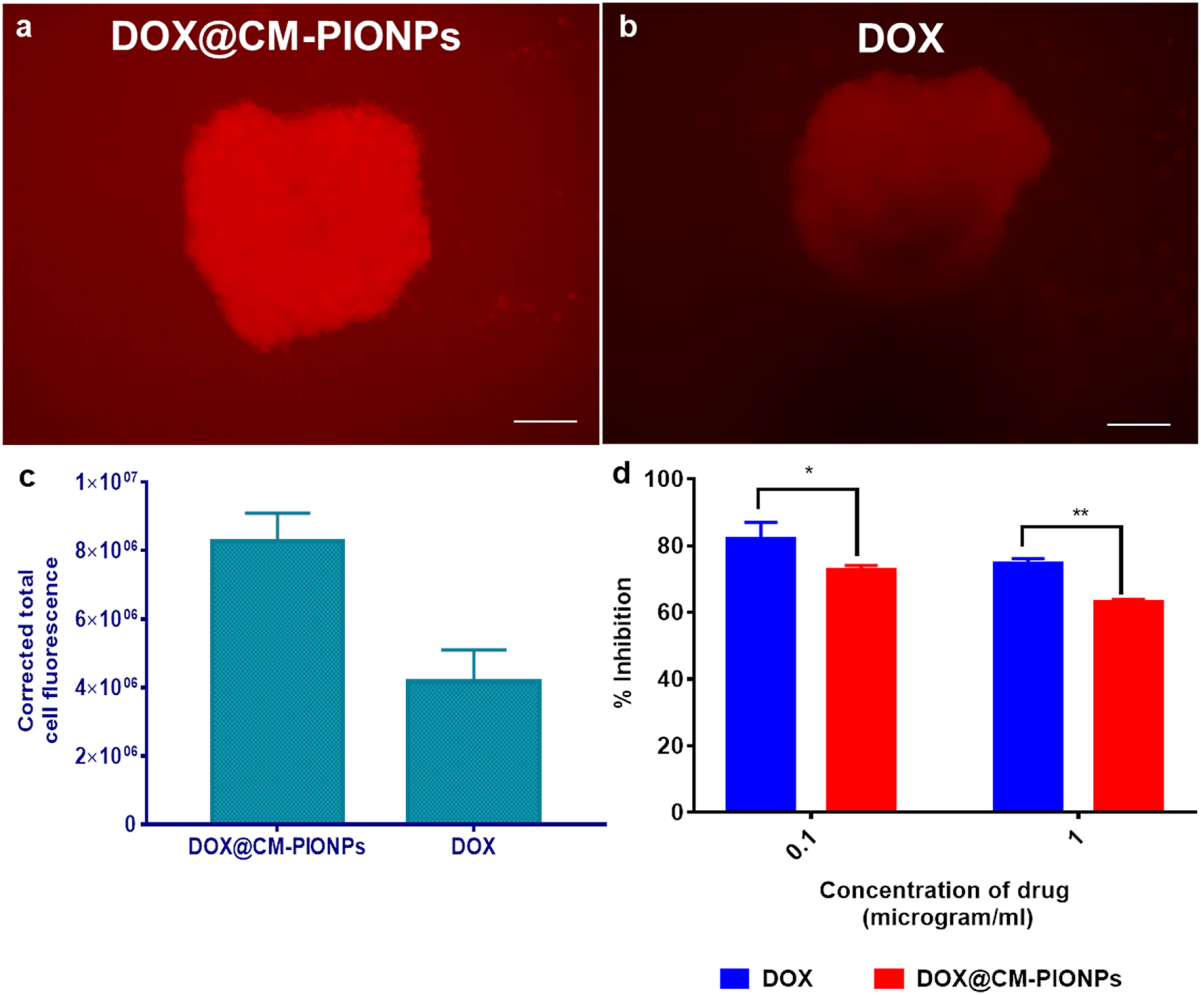
Tumoroid penetration study in GB-spheroids. Fluorescent microscopic images of representative GB-spheroids treated with (**a**) DOX@CM-PIONPs and (**b**) DOX. (**c**) Quantitative analysis of penetration potential. Total cell fluorescence of cells treated with DOX@CM-PIONPs were found to be significantly higher compared to DOX; (**d**) Cell viability analysis in GB-spheroids treated with DOX and DOX@CM-PIONPs. The results clearly show the effect of DOX@CM-PIONPs towards spheroids, where the free drug showed only less potency in reducing cell viability. The experiments were carried out in triplicates and error bars denote standard deviations.

The uptake efficiency of the particles were further investigated by fluorescence microscopy. The intensity of red fluorescence was significantly higher in cells treated with nanoparticles (Fig. 4a–c). Spheroids mimic tumor tissue architecture with limited drug penetration since the drugs were restricted to the outer monolayer of cells^42,43^. The effect of the drug to overcome the resistance formed as a result of spheroid formation was analyzed using cell viability assay after the treatment. Over the time frame of 24 h, cell death was significantly decreased in groups treated with nanoparticles compared to free drugs. The behaviour of DOX@CM-PIONPs against GB-spheroids exhibited 28–39% decrease in cell proliferation with increasing concentrations while doxorubicin can only cause 18–22% decrease in cellular proliferation when administered as free drug (Fig. 4d). A higher dosage of drug is required for the enhanced cell death due to the difficulty in penetration within the tumoroids. However, significant cell death was exhibited by NPs in 3-D culture compared to the un-encapsulated drug. In the present study, the synthesized nanoparticles tend to enter the inner core of the spheroids effectively decreasing cell growth. Thus, DOX@CM-PIONPs was found to be more effective in treating cancer spheroids.

### Toxicological evaluation in healthy BALB/c mice

BALB/c mice exposed to DOX@CM-PIONPs were monitored intermittently during the first 24 h, and thereafter daily for 14 days. None of the nanoparticles treated mice exhibited loss of body weight throughout the experiment. Most of the hematological parameters were found to be unchanged in all the treatment groups upon comparison with the control group (Supplementary Table 6).

Iron overload due to the degradation of IONPs has also been presumed to cause hepatotoxicity in animal models. Several adverse effects such as membrane degradation, generation of ROS, induction of apoptosis and DNA damage have been reported in IONPs administered murine models. We evaluated the level of oxidative stress in mice treated with DOX@CM-PIONPs using lipid peroxidation and reduced glutathione assay. Lipids are the major site of ROS alteration resulting in peroxide formation. Lipid peroxide levels in liver tissue homogenates of treated animals were measured. The mean value of lipid peroxidation was found to be 0.08 ± 0.71 (Day 3), 0.07 ± 0.07 (Week 1) whereas in the control group it shows only 0.08 ± 0.17 (Figure S19). No changes were observed both in lipid peroxide and in GSH concentrations in the group treated with DOX@CM-PIONPs when compared to control non treated group, whereas a slight increase in GSH level was obtained on Day 3 treated groups that were found to be insignificant on the comparison (Figure S20).

We have observed a slight upsurge in lipid peroxidation in the treated group when compared to control suggesting that NPs did not induce peroxide formation on prolonged exposure^44^ CM-PST coating did not allow interaction of lipids with the iron oxide, but prevented aggregation and increased the available reactive surface. The levels of GSH increased in tissues that exhibited higher levels of oxidative stress. Our investigation revealed that there was no significant increase in GSH level in CM-PST-IONPs treated BALB/c mice compared to non-treated mice.

### Therapeutic efficacy on the murine tumor model

**Figure 5:**
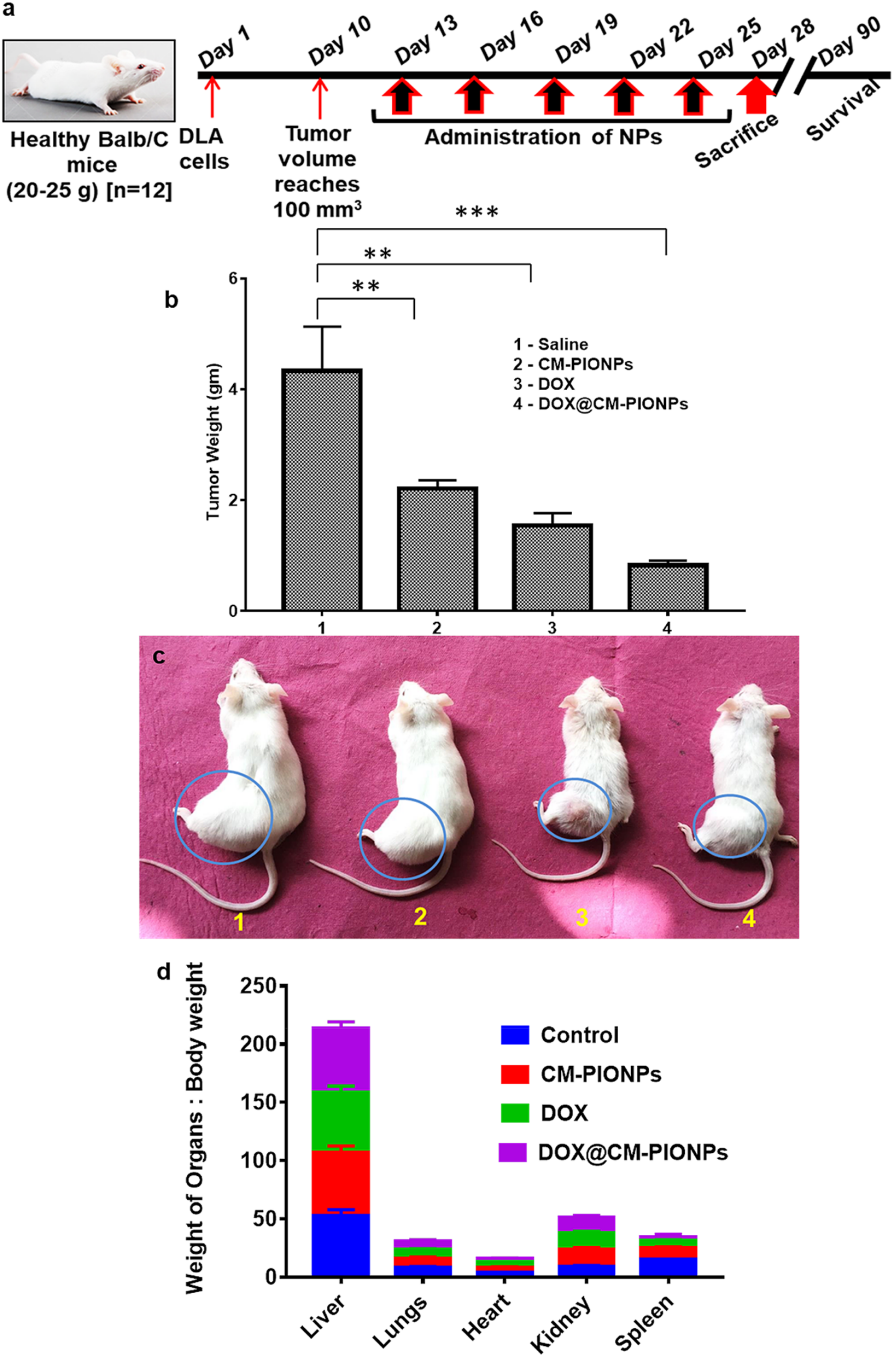
(**a**) Schematic representation of in vivo tumor reduction study. The treatment was started after 10 days of subcutaneous inoculation of DLA cells. The administration of compounds was carried out with an interval of 3 days for 15 days (reprinted from https://www.criver.com). (**b**) Tumor weight analysis. (**c**) Representative images of mice treated with CM-PIONPs, DOX and DOX@CM-PIONPs; (**d**) Organ-body weight ratio indicates an increased weight of spleen after 15 days of treatment in the control group is significantly found to be reduced in DOX@CM-PIONPs treated mice.

The encouraging observation from the in vitro cytotoxicity studies in 2-D and 3-D culture models urged us to investigate the in vivo antitumor potential of DOX@CM-PIONPs. Dalton Lymphoma Ascites (DLA) tumor model is an established syngeneic tumor model^20^. Here we had used a lower concentration of nanoparticles (equivalent dose of 2.5 mg/kg DOX) to avoid non-specific bio-distribution and systemic toxicity. Evaluation of various parameters on DLA solid tumor-bearing mice on 28 and 90 days of administration revealed a significant reduction in the tumor volume (*p* < 0.001) in groups administered with DOX@CM-PIONPs, DOX, and CM-PIONPs (Fig. 5a). Compared to all other groups, DOX@CM-PIONPs generated the best overall response. Representative photographic image of mice with different treatments is shown in Fig. 5b,c. The organ-body weight ratio indicated a reversal in the increased weight of spleen after 15 days of treatment in the control group with DOX@CM-PIONP administration (Fig. 5d, Fig. S21). Although free drug reduces the tumor burden, a significant increase in life span was observed in mice treated with DOX@CM-PIONPs. As expected in the control group, the mass of solid tumor amplified as the days advanced and the tumor burden was much greater compared to the DOX@CM-PST-IONPs treated mice. The median survival time (Figure S22) in the DOX@CM-PIONPs-treated group was longer (50 days) upon compariaion with the DOX-treated (45 days), CM@PIONPs-treated (40 days) and vehicle control group (20 days), suggesting that DOX@CM-PIONPs was more effective to prolong the lifespan by reducing the tumor burden. The efficiency of the particles in reducing tumor burden is appreciable, particularly considering the synergistic effect of doxorubicin and ROS provides a combinatorial effect for the complete annihilation of the tumor.

## Materials and method

### Ethics statement

All experimentation protocols were reviewed and approved by Institutional Review Board (IRB) of Regional Cancer Centre, Thiruvananthapuram, Kerala, India (IRB No.07/2015/03 dated 30/07/2015). All methods were carried out in accordance with the guidelines and regulations.

Experiments involving human blood samples was approved by the Institutional Human Ethics Committee, Regional Cancer Centre (HEC No. Nil dated 10/06/2015). Informed consent has been obtained from the healthy volunteer from our laboratory before sample collection.

Animal experiments were approved by the Institutional Animal Ethics Committee Regional Cancer Centre, according to the norms of Committee for the Purpose of Control and Supervision of Experiments on Animals (CPCSEA, Ministry of Fisheries, Animal Husbandry and Dairying, Department of Animal Husbandry and Dairying, Government of India,) (IAEC/RCC.No.2/15 (Ext.1) dated 05/10/2019). The study was carried out in compliance with the ARRIVE guidelines.

### Chemicals and reagents

Doxorubicin HCl (min 98% purity) was purchased from Carbosynth, Berkshire, UK. Monochloroacetic acid was purchased from Sigma Aldrich. Tissue culture reagents including Dulbecco’s modified Eagle medium (DMEM) and Phosphate-buffered Saline (PBS) were purchased from Hyclone, GE Healthcare Life Sciences. Antibiotic–antimycotic mixture was purchased from Gibco Life Technologies Corporation, USA. All chemicals were purchased from Merck chemicals, India.

### Carboxymethylation of PST001

Isolation and characterization of PST001 is described in section SI-1 in the supplementary information. Carboxymethylation of PST001 was carried out as described earlier with slight modifications^23^. Briefly, PST001(5.5 mg/ml) dissolved in distilled water followed by the addition of sodium hydroxide (0.6 mg/ml) and kept in a water bath for 60 min at 60 °C. Chloroacetic acid (8.5 mg/ml) was added and kept at 70 °C for 2 h. After incubation, the CM-PST formed was precipitated with 70% methanol and centrifuged at 8000 rpm for 10 min. The pellet was washed twice with 100% methanol and then with 100% ethyl alcohol. The pellet was kept for dialysis against distilled water for 24 h to remove excess reagents and was lyophilized. The final product was stored at 4 °C until further use. The formation of CM-PST was confirmed by FTIR (Thermo Nicolet, Avatar 370) and the degree of substitution was calculated as described in section SI-2.

### Fabrication of biopolymer capped IONPs

Iron oxide nanoparticles were synthesized by chemical co-precipitation methods followed by APTES coating as reported earlier with slight modifications^24^. For a reaction volume of 50 ml, 250 mg IONPs obtained from co-precipitation of ions were dispersed in 49 ml methanol and sonicated for 30 min and 1 ml APTES was mixed under mechanical stirring. The solvent was removed by evaporation and the aminated IONPs obtained were dispersed in water. The aminated iron oxide nanoparticles were allowed to react with CM-PST (10 mg/ml) under sonication for 30 min followed by magnetic stirring for 24 h. The particles were magnetically separated and dissolved in deionized water. The surface morphology and size were evaluated using transmission electron microscopy (TEM-Jeol/JEM 2100). The stability, hydrodynamic size, and polydispersity index (PI) was determined using dynamic light scattering (DLS) (HORIBA, Japan). The protocol for analyzing the biocompatibility of the particles is described in section SI-3.

### Doxorubicin loading and pH-responsive DOX release kinetics of DOX@CM-PIONPs

For drug loading, we have employed three routes to confirm the role of CM-PST in efficient encapsulation of doxorubicin. In route ‘a’, 10 mg IONPs dissolved in water was stirred with 100 µg/ml doxorubicin hydrochloride for 24 h at 37 °C while in route ‘b’, 10 mg aminated IONPs dissolved in water was allowed to react with 100 µg/ml doxorubicin hydrochloride for 24 h at 37 °C. In route ‘c’, 10 mg CM-PIONPs dissolved in water was stirred with 100 µg/ml doxorubicin hydrochloride for 24 h at 37 °C. After all the preparations, the solutions were kept for dialysis for 24 h and magnetically separated. The obtained particles were dissolved in distilled water and physically characterized by DLS. Next, encapsulation efficiency was measured in DOX@CM-PIONPs using HPLC analysis after the digestion of the particles using concentrated HCl^41^.

The release kinetics of the drug from DOX@CM-PIONPs were studied at 37 °C in two different buffers at pH 7.4 (phosphate buffer), and 6.5 (phosphate buffer) by dialysis method. At time intervals of 1–72 h, release buffer (1 ml) was replaced with an equal volume of fresh buffer and the concentration of drug was determined by HPLC^41^.

### Culture and maintenance of cell lines

Human lung adenocarcinoma (A549), human glioblastoma (U-87MG), and murine melanoma (B16F10) were procured from NCCS, Pune, India. Cells were maintained in Dulbecco’s modified Eagle medium (DMEM) with 10% fetal bovine serum (FBS) and 5% CO_2_ at 37 °C.

### In vitro cytotoxicity assay

The growth inhibitory capacity of the DOX@CM-PIONPs was evaluated in the above panel of cancer cell lines by MTT (3-[4,5-dimethylthiazol-2yl]-2,5 diphenyltetrazolium)assay as previously reported^11^. In brief, cells (10,000 cells/well) were seeded in a 96-well tissue culture plate and incubated 24 h for cell attachment. The particles with varying concentrations were treated against cell lines for 24, 48 and 72 h. After the prescribed incubation time, the media was discarded and MTT dye (1 mg/ml) was added to each well. After 3 h, the formazan crystals were dissolved in MTT lysis solution and absorbance was measured at 570 nm using a microplate spectrophotometer (BioTek, Power Wave XS). The proliferation rate and inhibitory rate of the cells were calculated with the following formulae:

$$ \begin{aligned} {\text{Proliferation rate}}\;\left( {{\text{PR}}} \right)\% & = \left[ {\left( {\left( {{\text{Abs sample}} - {\text{Abs blank}}} \right)} \right)/\left( {\left( {{\text{Abs control}} - {\text{Abs blank }}} \right)} \right)} \right] \times 100 \\ {\text{Inhibitory rate}}\;\left( {{\text{IR}}} \right)\% & = 100 - {\text{PR}} \\ \end{aligned} $$

Cell permeability as a marker of apoptosis was evaluated in treated cells using the acridine orange–ethidium bromide dual staining procedure. These experiments were performed as described earlier^11^. Briefly, cells (10,000 cells/well) were seeded in a 96-well tissue culture plate and incubated with the particles for 48 h. The treated cells were observed under an inverted fluorescent microscope, using a FITC filter (Olympus BX51, Singapore) to view the apoptotic and non-apoptotic cells^11^.

### Cellular uptake studies of DOX@CM-PIONPs

For qualitative analysis, cells were seeded on 24-well tissue culture plates at a seeding density of 1 × 10^5^ cells. After 24 h, the adherent cells were washed twice with PBS followed by replacement of media containing DOX@CM-PIONPs containing 5 μg/ml DOX and incubated for 3 h at 37 °C. Cells were washed with PBS, fixed with 4% paraformaldehyde for 20 min, and counterstained with DAPI for 7–8 min. After PBS washing, the fixed cells were imaged under an inverted fluorescent microscope (Olympus BX51, Singapore)^41^.

For flow cytometric analysis, cells were seeded on 24-well tissue culture plates at a seeding density of 1 × 10^5^ cells. After 24 h, the adherent cells were washed twice with PBS followed by replacement of media containing DOX@CM-PIONPs containing 5 μg/ml DOX and incubated for 3 h at 37 °C. Cells were washed with PBS, trypsinized and particle uptake was analyzed in the red channel in the flow cytometry. Data were collected from > 10,000 gated events, using the BD FACS software program (BD Biosciences, USA)^41^.

For quantitative analysis, cells were seeded on 24-well tissue culture plates at a seeding density of 1 × 10^5^ cells. After 24 h, the adherent cells were washed twice with PBS followed by replacement of media containing DOX@CM-PIONPs containing 5 μg/ml DOX and incubated for 3 h at 37 °C. Cells were washed with PBS, lysed with RIPA lysis buffer and protein was precipitated using acetonitrile. After precipitation, the concentration of the drug in the supernatant was determined using HPLC analysis^41^.

### Determination of ROS using DCFH-DA assay

Cells seeded in 96-well plates were incubated with the nanoparticles at a fixed concentration of 0.5 mg/ml for 24 h. After incubation, cells were washed with PBS and allowed to incubate with carboxy-2′,7′-dichloro-dihydro-fluorescein diacetate (10 µM) for 30 min in dark. Then, cells were lysed using lysis buffer and transferred to 96-black well microplate. The fluorescence intensiy of cell lysate was measured using spectrofluorimeter (Flx800 BioTek, USA) with excitation and emission. For qualitative analysis, the cells were observed under an inverted fluorescent microscope, using an FITC filter (Olympus BX51, Singapore).

### Mechanism of cellular uptake of nanoparticles

The endocytosis pathway involved in the uptake of nanoparticles in A549 cells was determined as previously described. Briefly, 10^6^ cells/well were seeded and pre-incubated for 1 h with the endocytic inhibitors (chlorpromazine HCl, Cytochalasin D, Methyl β cyclodextrin and Amiloride HCl) in serum-free medium at 37 °C. Cells were treated with nanoparticle with a drug concentration of 5 µg/ml after 1 h and incubation continued for another 2 h at 37 °C. The cell lysate was precipitated by acetonitrile and collected supernatant analyzed by HPLC^41^.

### Establishment and treatment of 3-D tumor glioblastoma model

Human malignant glioblastoma cells U87MG were cultured in DMEM supplemented with 10% FBS and 1X antibiotic at 37 °C in a humid incubator with 5% CO_2_. Cells were then trypsinized and counted the number of cells using a hemocytometer. Cells were seeded in a 96-well culture plate at a cell density of 2 × 10^4^ cells/well to form the control 2-D monolayers. Cells were also seeded on the agarose coated tissue-culture plates at a cell density of 2.0 × 10^4^ cells/well for 6.9 mm microwells. Cells began to aggregate within 4 h and formed into glioma spheroids within 24 h of incubation. The formed spheroids were imaged under a phase-contrast microscope (Olympus BX51, Singapore) for 4 days. The spheroid diameter was measured using ImageJ software.

After 96 h of incubation, the spheroids were stained using Calcein AM staining followed by capturing the microscopic image using an inverted fluorescent microscope, using a FITC filter (Olympus BX51, Singapore). The intracellular uptake efficiency of the nanoparticles and drug in the spheroid model was also analyzed. Briefly, the spheroids were treated with doxorubicin and DOX@CM-PST-IONPs at a drug concentration of 1 µg/ml for 6 h. After incubation, the cells were imaged using a fluorescent microscope and corrected total cell fluorescence (CTCF) was measured using ImageJ software.

The cell viability of treated cells was measured using the MTT assay after the treatment of spheroids. In brief, the spheroids were treated with doxorubicin and DOX@CM-PST-IONPs at a drug concentration of 0.1 µg/ml and 1 µg/ml for 24 h. After incubation, the spheroids were mechanically disintegrated using gentle pipetting followed by its incubation with MTT dye (1 mg/ml). After 3 h of incubation, the formazan crystals so formed were dissolved in lysis buffer and the OD was read at 570 nm.

### In vivo evaluation

All the animal experimentation procedures described including maintenance were reviewed and approved by the Institutional Animal Ethics Committee according to the norms of Committee for the Purpose of Control and Supervision of Experiments on Animals (CPCSEA, Ministry of Fisheries, Animal Husbandry and Dairying, Department of Animal Husbandry and Dairying, Government of India,). The procedure for toxicological evaluation is described in section SI-4 and sub-acute toxicity analysis is represented in supplementary Scheme 1 of the supporting information. The study was performed in accordance with ARRIVE guidelines.

### Animal preparation and experimental design

Forty-eight female BALB/c mice (25–30 g, 6–8 weeks old) were randomly divided into four groups.

Group 1. Control group (n = 12): intraperitoneal administration of PBS (0.5 ml) for five days (3 day interval in 15 days). After 15 days, the treated rats (n = 6) were euthanized using over dose of anaesthesia and tumor weight was measured. The reminaing mice (n = 6) were kept for survival analysis.

Group 2. CM-PIONPs treated group (n = 12): intraperitoneal administration of CM-PIONPs (10 mg/kg day) (0.5 ml) for five days (3 day interval in 15 days). After 15 days, the treated rats (n = 6) were euthanized using over dose of anaesthesia and tumor weight was measured. The reminaing mice (n = 6) were kept for survival analysis.

Group 3. DOX treated group (n = 12): intraperitoneal administration of DOX (2.5 mg/kg day) (0.5 ml) for five days (3 day interval in 15 days). After 15 days, the treated rats (n = 6) were euthanized using over dose of anaesthesia and tumor weight was measured. The reminaing mice (n = 6) were kept for survival analysis.

Group 4. DOX@CM-PIONPs treated group (n = 12): intraperitoneal administration of DOX@CM-PIONPs (equivalent dose of DOX) (0.5 ml) for five days (3 day interval in 15 days). After 15 days, the treated rats (n = 6) were euthanized using over dose of anaesthesia and tumor weight was measured. The reminaing mice (n = 6) were kept for survival analysis.

The development of the sub-cutaneous Dalton Lymphoma Ascites (DLA) tumor model as described previously^45^.

### Statistical analysis

All experiments were conducted in triplicate and the results were expressed as the mean ± standard deviation. Student’s t-tests were used to analyze in vitro and in vivo data. All statistical analyses were performed using GraphPad Prism software version 5.0 (GraphPad Software, CA, USA) and Microsoft Excel 2010. Survival curves of the mice in each experiment were compared using Kaplan–Meier analysis with GraphPad Prism software version 5.0.

## Conclusion

In summary, the novel drug-loaded iron oxide nanoparticles investigated in this study not only demonstrated an excellent in vitro cytotoxicity in 2-D culture but also presented to be a promising biocompatible targeted nanocarrier delivery construct in the 3-D culture system. Tumor niche-specific drug release kinetics facilitated DOX@CM-PIONPs to accomplish superior constructive therapeutic effects. In the diagnostic aspect, the fluorescent drugs transformed the nanosystem to be an effective fluorescence probe for non-invasive imaging to monitor the cellular uptake and release of the permeated doxorubicin. Finally, the DOX@CM-PIONPs was explored in murine models that showed greater therapeutic efficiency grander to the clinically used DOX. Although further investigations are reasonable, the effect exhibited by DOX@CM-PIONPs holds an objective for future preclinical and clinical applications in tumor management.

## Supplementary Information

Supplementary Information
