# Supplementary material for: A comprehensive study on 2D, 3D and solid tumor environment to explore a multifunctional biogenic nanoconjugate
Source: Sci Rep. 2021 Apr 22;11:8721. doi: 10.1038/s41598-021-87364-y (PMC8062514; doi:10.1038/s41598-021-87364-y)
Supplement: Supplementary file 1 — Supplementary Information [file 41598_2021_87364_MOESM1_ESM.docx]

**A comprehensive study on 2D, 3D and solid tumor environment to explore a multifunctional biogenic nanoconjugate**

**Unnikrishnan BS^1^, Preethi G U^1^, Sreelekha T T^1^***

**^1^**Laboratory of Biopharmaceuticals and Nanomedicine, Division of Cancer Research, Regional Cancer Centre (RCC), Thiruvananthapuram-695011, Kerala, India

**Supplementary Information**

| **Contents** | **Page No** |
| --- | --- |
| Data SI:1-SI:4 | 1-2 |
| Scheme S1 | 3 |
| Table S1-S6 | 4-7 |
| Figures S1-S22 | 8-23 |

**SI:1: Isolation of polysaccharide PST001 from the Tamarind seed kernel**

Polysaccharide (PST001) was isolated from the seed kernel of *Tamarindus indica* as reported earlier from our laboratory [1]. 100 gm of fine powdered seed kernel powder was boiled in 1L distilled water with constant stirring. The hot solution was kept for cooling and the polysaccharide in the supernatant obtained after centrifugation was precipitated using ethanol. The precipitate obtained was dissolved in distilled water and protein was separated in a funnel using chloroform. The aqueous layer was dialyzed against water and the lyophilized. The isolated polysaccharide was analyzed with high-performance liquid chromatography (Shimadzu, Japan) using GPC/SEC columns (Agilent Technologies, USA).

**SI:2: Determination of the Degree of Substitution (DS)**

The back titration method was used to determine the DS. Five grams of sample was dispersed in water using a magnetic stirrer. 15 ml of 6M HCl was added to the suspension and stirred for 1 hour. The dispersion was filtered and washed with methanol (80%) until the solution becomes neutral and dried at 60^0^C. About 500 mg dissolved in 20 ml 0.2 M NaOH and diluted to 100 ml with distilled water. The product (25 ml) was back titrated with 0.05 M HCl using phenolphthalein as the indicator and calculated the rate of carboxymethylation [2].

**SI:3: Biocompatibility assay of DOX@CM-PIONPs**

For hemolysis assay, human RBC diluted in 0.9% saline was gently mixed with nanoparticles and incubated for 2 hours. The positive control was saline while the negative control was distilled water. After incubation, the sample was centrifuged and absorbance (abs) of the supernatant was measured at 541 nm. The percentage of hemolysis was calculated [3].

**SI:4: Toxicological evaluation in Balb/C mice**

Acute toxicity of CM-PIONPs was evaluated in BALB/C mice weighing about 25-30 g. Throughout the study period, animals were monitored for the development of any toxicological signs and symptoms such as abnormal posture, abnormal movements, difficulties in respiration, and changes in body weight and feed intake. Animals were euthanized using over dose of anaesthesia and gross necropsies were performed which include the examination of the external surface of the body and internal organs.

**Preparation of liver homogenate**

For lipid peroxidation studies, 10% liver tissue homogenate (Phosphate buffer, pH 7.4) was prepared from the untreated and treated animals. The homogenate was then centrifuged at 3500 rpm for 10 min at 4°C in a refrigerated centrifuge. The resultant supernatants were maintained in an ice bath and are used as the sample for the estimation of total protein and lipid peroxidation.

**Lipid Peroxidation (LPO) Assay**

This method is based on the increase in malondialdehyde formation in response to increased peroxides within the cell. The malondialdehyde (MDA) produced reacts with thiobarbituric acid reactive substances (TBARS) forming a complex. The amount of MDA formed was measured spectrophotometrically at 532 nm.

**Glutathione assay**

This method is used to estimate the amount of glutathione (GSH) for the measurement of oxidative stress in healthy tissues. Tissue homogenate (0.5 ml) was allowed to react with 125 µl of 25% Trichloroacetic acid (TCA) to precipitate proteins. The tubes were cooled in ice for 5 minutes and the mixture was further diluted with 0.6ml of 5% TCA and centrifuged for 10 minutes and 0.3ml of supernatant was taken for GSH estimation. The volume of aliquot was made up to 1 ml with 0.2M Phosphate buffer and 2ml of freshly prepared 0.6 mM DTNB was added to the tubes. The intensity of the yellow color was read at 412 nm.

**Supplementary scheme 1: Sub-acute toxicity study:**

Twenty-four healthy BALB/c mice (25-30 g, 6–8 weeks old) were randomly divided into four groups.

Group 1. Control group (n = 6): intraperitoneal administration of PBS (0.5 ml) for fourteen days. After 15 days, the treated rats (n=6) were euthanized using over dose of anaesthesia and blood was collected by cardiac puncture.

Group 2. CM-PIONPs treated group (n = 6): intraperitoneal administration of CM-PIONPs (10 mg kg-1 d-1) (0.5 ml) for fourteen days. After 15 days, the treated rats (n=6) were euthanized using over dose of anaesthesia and blood was collected by cardiac puncture.

Group 3. DOX treated group (n = 6): intraperitoneal administration of DOX (2.5 mg kg-1 d-1) (0.5 ml) for fourteen days. After 15 days, the treated rats (n=6) were euthanized using over dose of anaesthesia and blood was collected by cardiac puncture.

Group 4. DOX@CM-PIONPs treated group (n = 6): intraperitoneal administration of DOX@CM-PIONPs (equivalent dose of DOX) (0.5 ml) for fourteen days. After 15 days, the treated rats (n=6) were euthanized using over dose of anaesthesia and blood was collected by cardiac puncture.

**
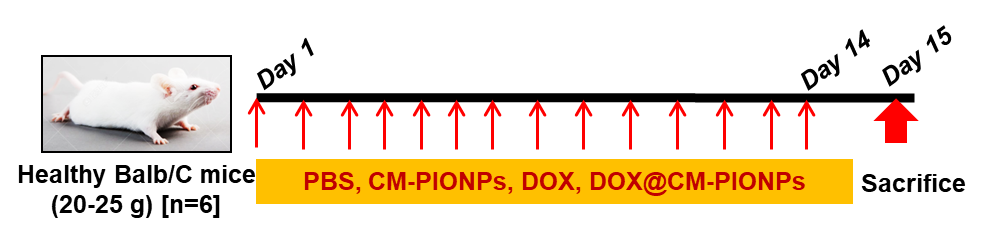
**

**Supplementary Table**

**Supplementary Table1: Physiological parameters of the modified PST001**

| **Parameters** | **PST001** | **CM-PST** |
| --- | --- | --- |
| **pH** | **6.8** | **5.77** |
| **Reaction with Sodium bicarbonate** | **No Reaction** | **Brisk effervescence** |

**Supplementary Table 2: Physical characterization of particles synthesized by three routes**

| **Route** | **Hydrodynamic size** | **Zeta potential** | **PI** |
| --- | --- | --- | --- |
| **a** | 310.6 nm | -0.6 mV | 5.118 |
| **b** | 380.6 nm | +20 mV | 2.318 |
| **c** | **213.6 nm** | **-7.9 mV** | **0.594** |

**Supplementary Table 3: Stability Study**

| **Nanoformulation** | **Duration of storage**  **at 4°C in liquid form** | **Zeta potential (mV)** | **Hydrodynamic size (nm)** |
| --- | --- | --- | --- |
| **CM-PIONPs** | 30 days | -6.5 | 324.5 |
|  | 60 days | -7.2 | 360.8 |
|  | 120 days | -6.8 | 402.8 |
| **DOX@CM-PIONPs** | 30 days | -7.4 | 225.8 |
|  | 60 days | -6.8 | 280.1 |
|  | 120 days | -5.8 | 305.2 |

**Supplementary Table 4: Mean Absorbance of MTT assay**

|  |  | **B16F10** | | **A549** | | **U87MG** | |
| --- | --- | --- | --- | --- | --- | --- | --- |
| **Incubation Period** | **Concentration (microgram/ml)** | **DOX** | **DOX-CM@PIONPs** | **DOX** | **DOX-CM@PIONPs** | **DOX** | **DOX-CM@PIONPs** |
| **24 h** | 0 | 1.033 | 1.033 | 0.919 | 0.919 | 0.586 | 0.586 |
|  | 0.001 | 1.27547 | 1.150184 | 0.833441 | 0.741118 | 0.480286 | 0.444833 |
|  | 0.01 | 1.21495 | 0.969171 | 0.780691 | 0.669404 | 0.445067 | 0.37293 |
|  | 0.1 | 1.06666 | 0.857584 | 0.734557 | 0.499778 | 0.384709 | 0.354921 |
|  | 1 | 0.91504 | 0.586929 | 0.685206 | 0.199473 | 0.354823 | 0.338815 |
| **48 h** | 0 | 1.133 | 1.133 | 0.989 | 0.989 | 0.606 | 0.606 |
|  | 0.001 | 0.97336 | 0.951154 | 0.844112 | 0.780231 | 0.484012 | 0.433169 |
|  | 0.01 | 0.97177 | 0.81508 | 0.816024 | 0.501821 | 0.435047 | 0.366509 |
|  | 0.1 | 0.88215 | 0.730558 | 0.691014 | 0.333438 | 0.433169 | 0.344147 |
|  | 1 | 0.51483 | 0.46385 | 0.560565 | 0.197143 | 0.307787 | 0.316029 |
| **72 h** | 0 | 1.23 | 1.23 | 1.023 | 1.023 | 0.698 | 0.698 |
|  | 0.001 | 0.98154 | 0.92865 | 0.85175 | 0.803028 | 0.529852 | 0.451815 |
|  | 0.01 | 0.95140 | 0.86715 | 0.776866 | 0.347655 | 0.444207 | 0.403584 |
|  | 0.1 | 0.67367 | 0.62607 | 0.557228 | 0.229246 | 0.422779 | 0.351583 |
|  | 1 | 0.46125 | 0.293355 | 0.284087 | 0.148011 | 0.333784 | 0.32101 |

**Supplementary Table 5: HPLC analysis of Doxorubicin**

| **Concentration (microgram/ml)** | **Retention Time (min)** | **Area** | **Height** |
| --- | --- | --- | --- |
| 3.81 | 7.49 | 4645768 | 12559.9 |
| 7.632 | 7.647 | 1005896 | 26175.7 |
| 15.625 | 7.602 | 1949296 | 56407.9 |
| 31.25 | 7.587 | 3966374 | 108044.1 |
| 62.5 | 7.592 | 7886238 | 223473 |

**Supplementary Table 6: Haematological and biochemical analysis. Data were expressed in Mean ± SD.**

|  | **Control** | **CM-PIONPs** | **DOX** | **DOX@CM-PIONPs** |
| --- | --- | --- | --- | --- |
| **HAEMATOLOGY** | | | | |
| **Hb (g/dl)** | 14.7±0.3 | 13.2±0.1 | 12.4±0.5 | 14.2±0.1 |
| **TC (/cmm)** | 5500.0±103.0 | 6800.0±89.0 | 5100.0±121.0 | 6500.0±65.0 |
| **DC (%) Poly** | 24.0±1 | 28±1 | 25±1 | 24±1 |
| **Lymph** | 75.0±6.0 | 76.0±3.0 | 73.0±5.0 | 73.0±2.0 |
| **Eosin** | 2.0±1.0 | 2.0±1.0 | 2.0±1.0 | 3.0±1.0 |
| **Mono** | Nil | Nil | Nil | Nil |
| **Baso** | Nil | Nil | Nil | Nil |
| **Platelets (per cm^3^)** | 6,50,000.0±1500.0 | 5,10,000.0±1250.0 | 4,90,000.0±1000.0 | 5,70,000.0±1200.0 |
| **PCV (%)** | 50.0±0.0 | 42.0±0.0 | 43.0±0.0 | 52.0±0.0 |
| **BIOCHEMISTRY** | | | | |
| **RBS (mg/dl)** | 170.0±5.0 | 125.0±2.2 | 130.0±2.0 | 127.0±3.0 |
| **Urea (mg/dl)** | 41.0±0.3 | 44.0±0.5 | 51.0±0.5 | 48.0±0.4 |
| **Creatinine (mg/dl)** | 0.50±0.01 | 0.70±0.01 | 0.80±0.01 | 0.50±0.01 |
| **S. Bilirubin Direct (mg/dl)** | 0.02±0.00 | 0.02±0.00 | 0.02±0.00 | 0.02±0.00 |
| **SGOT (IU/L)** | 39.0±1.0 | 40.0±1.0 | 39.0±1.0 | 35.0±1.0 |
| **SGPT (IU/L)** | 41.0±2.0 | 44.0±1.0 | 37.0±1.0 | 38.0±1.0 |
| **Total Protein (g/dl)** | 5.0±0.  5 | 5.0±0.8 | 5.0±0.4 | 5.0±0.3 |
| **Albumin (g/dl)** | 2.0±0.9 | 2.0±0.9 | 2.0±0.7 | 2.0±0.6 |
| **Globulin (g/dl)** | 2.0±0.6 | 2.0±0.9 | 2.0±0.7 | 2.0±0.7 |

***Supplementary figure S1: FTIR analysis of CM-PST***


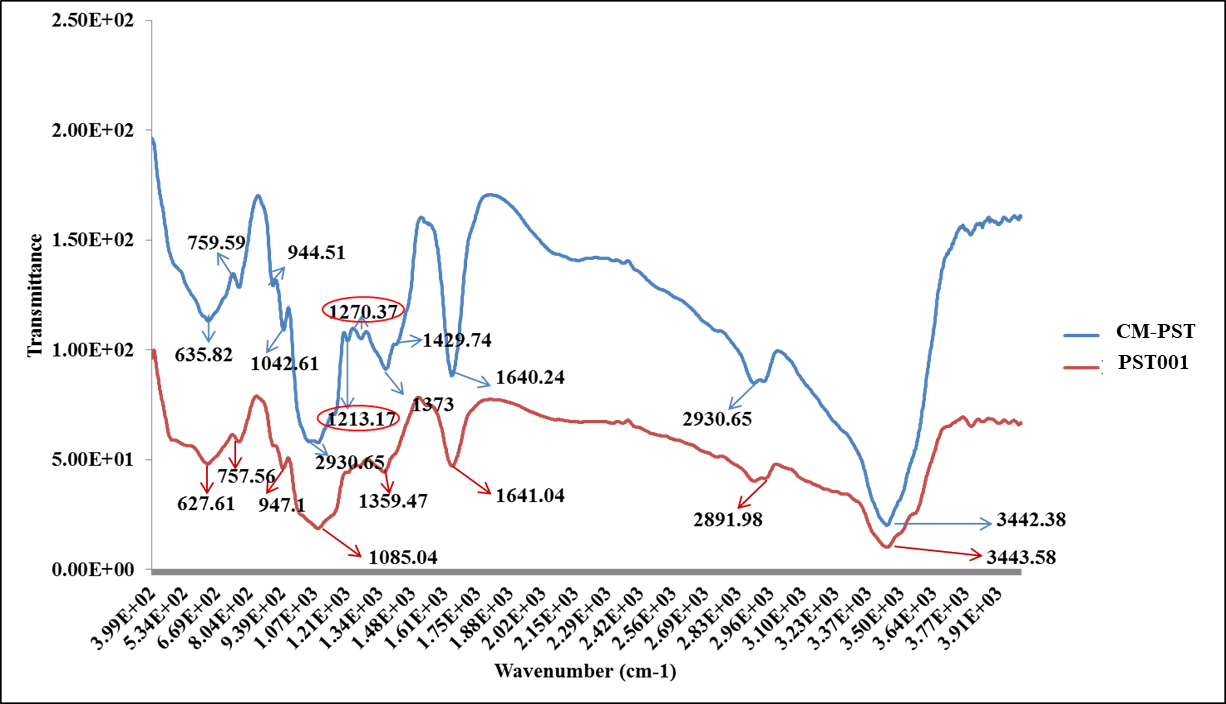


***Supplementary figure S2: TEM image of IONPs***

***
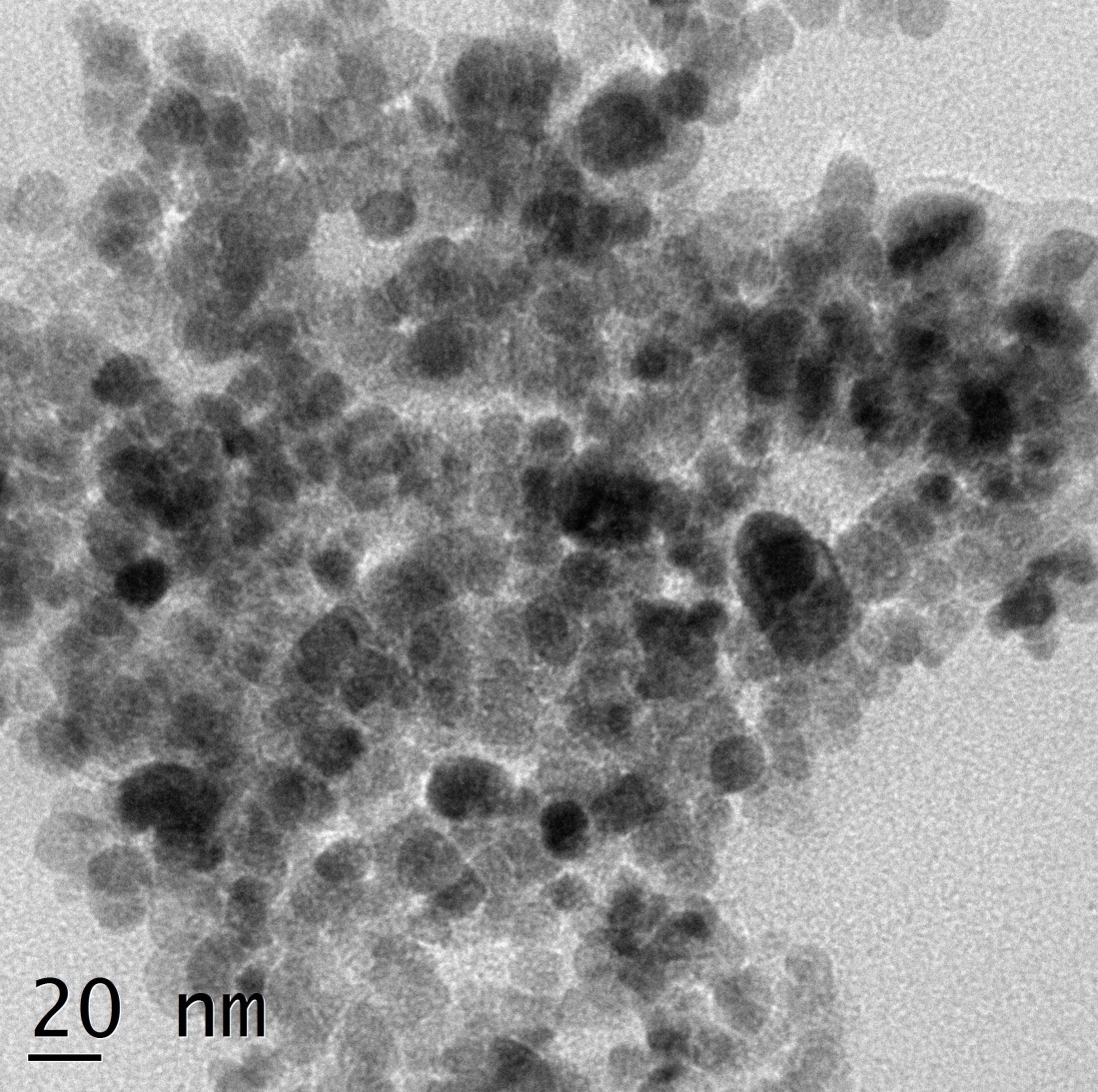
***

***Supplementary figure S3: FTIR analysis of CM-PIONPs***


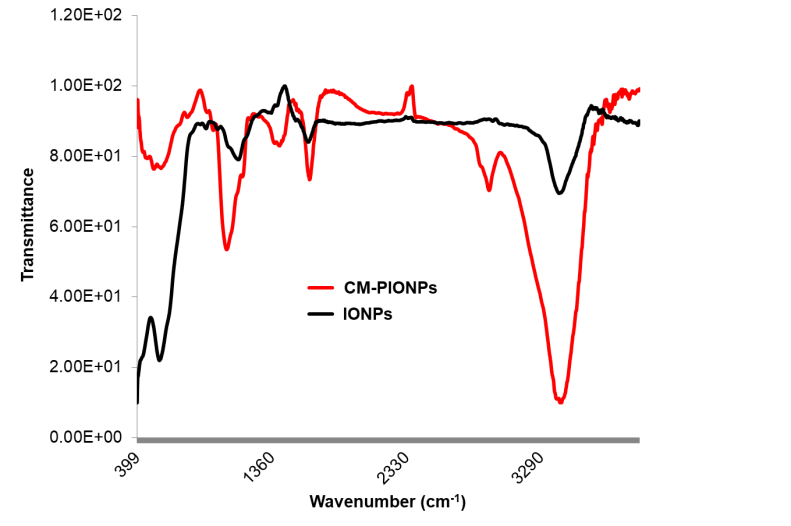


***Supplementary figure S4: Synthetic routes of DOX@CM-PIONPs***


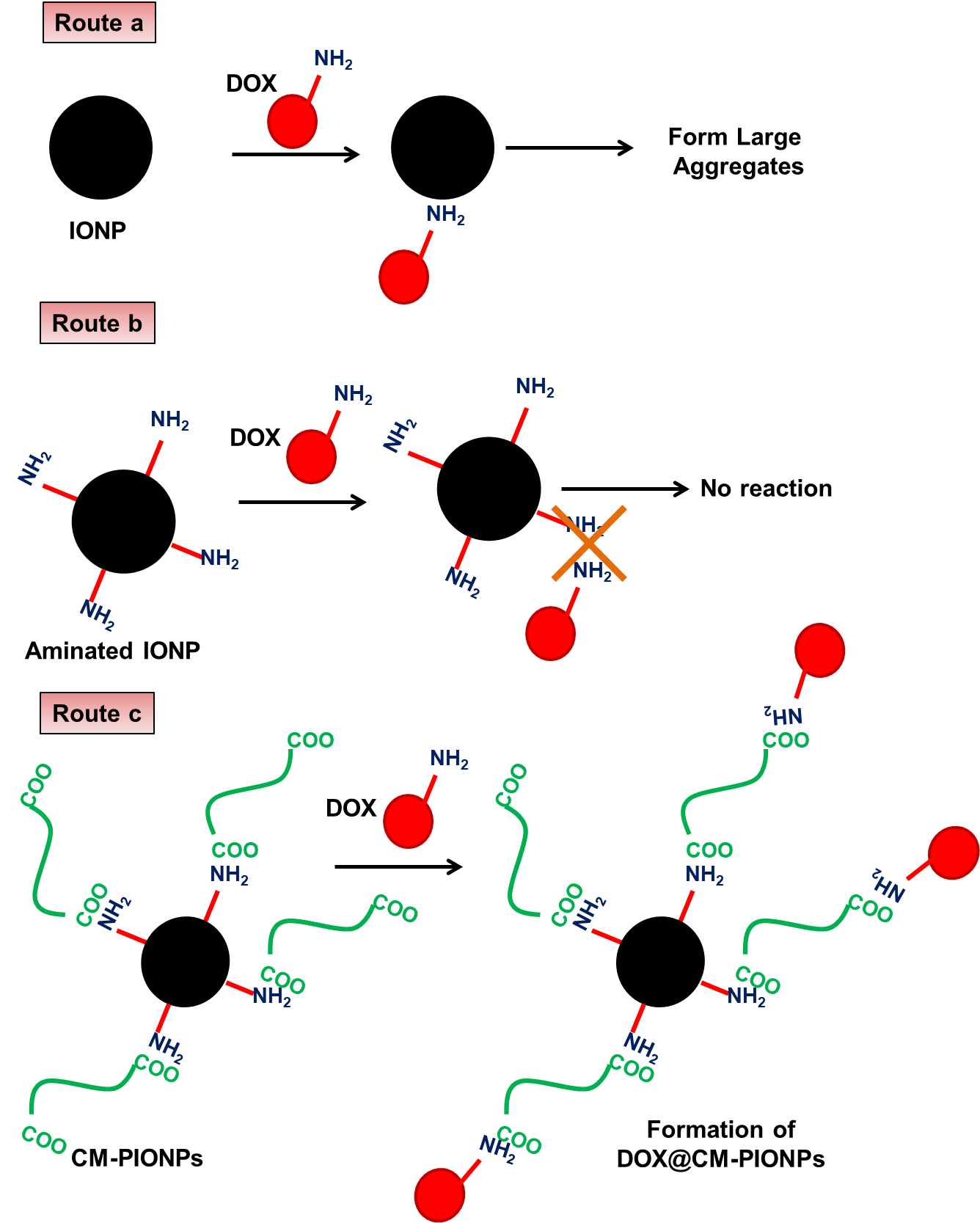


***Supplementary figure S5: SAED pattern of DOX@CM-PIONPs***


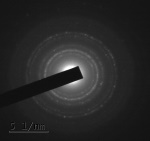


***Supplementary figure S6: DLS histogram of DOX@CM-PIONPs***

***
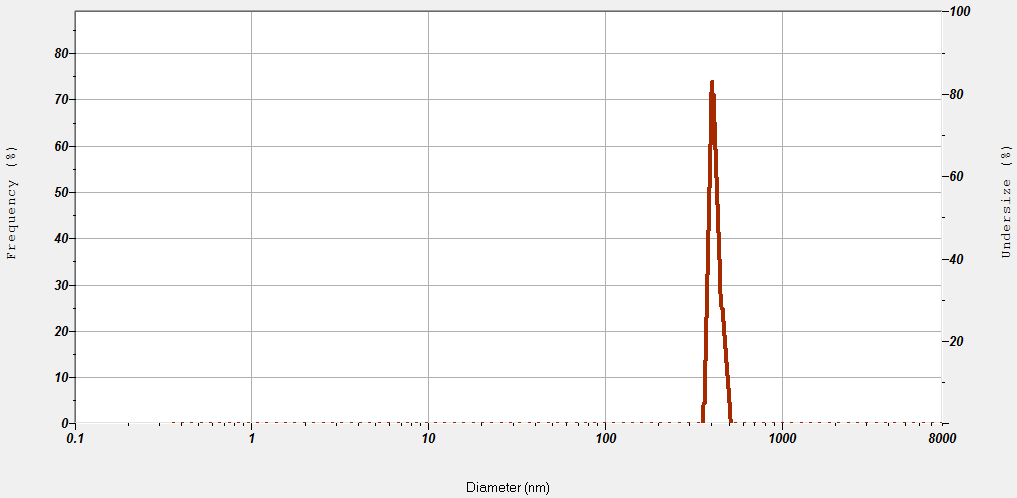
***

***Supplementary figure S7: Drug release kinetics of DOX@CM-PIONPs***


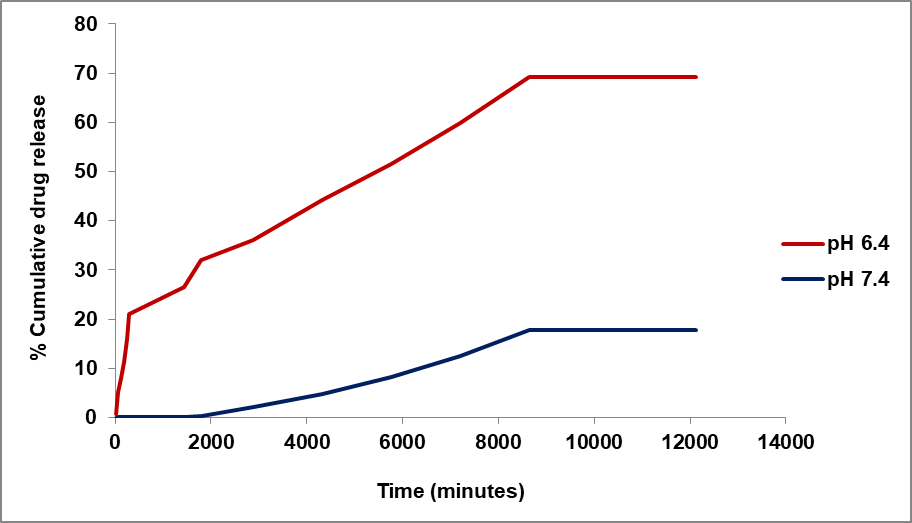


***Supplementary figure S8: Phase contrast microscopic images of RBCs incubated with CM-PIONPs & DOX@CM-PIONPs***


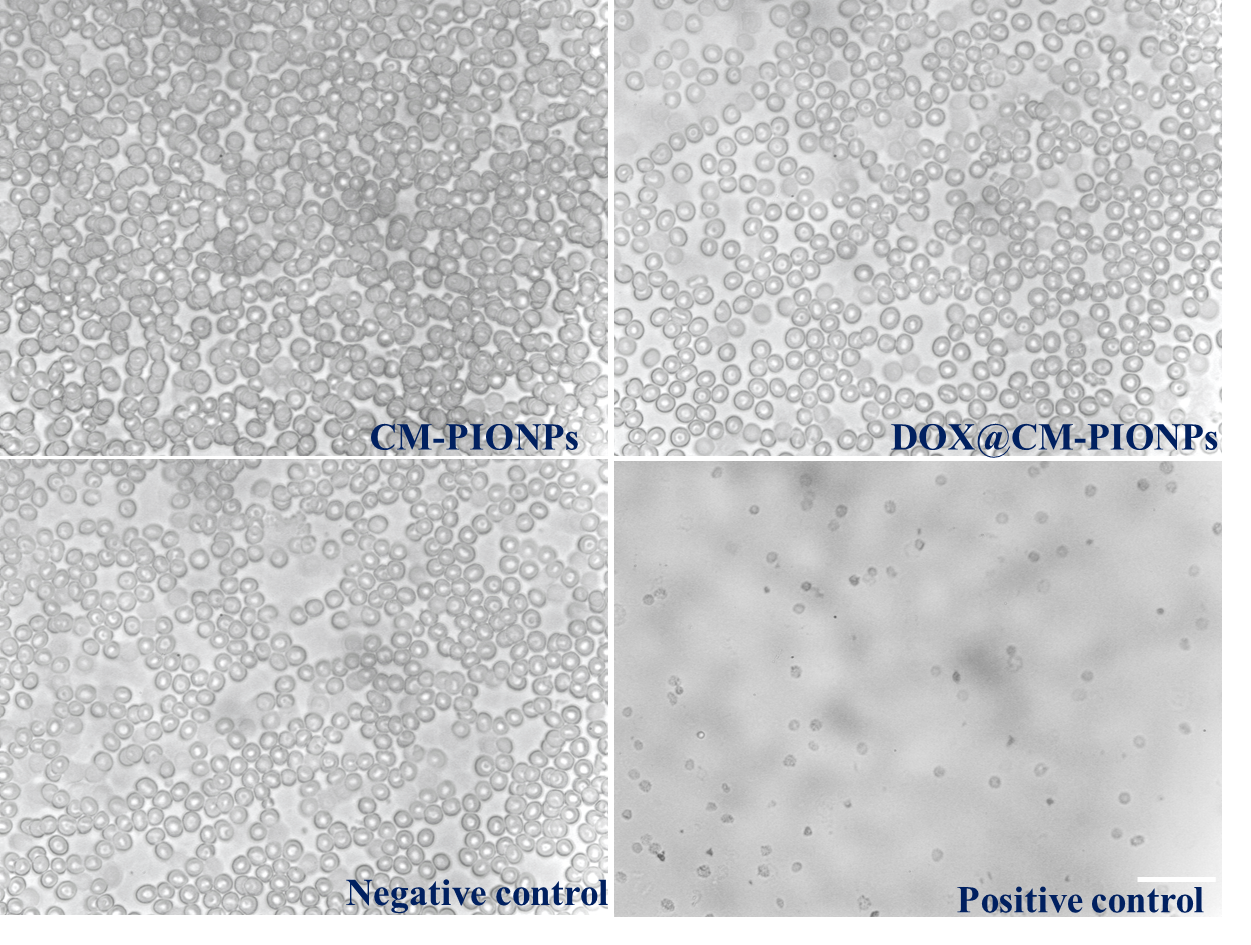


***Supplementary figure S9: Cell viability analysis of lymphocytes treated with CM-PIONPs for 24, 48 and 72 hours***

***
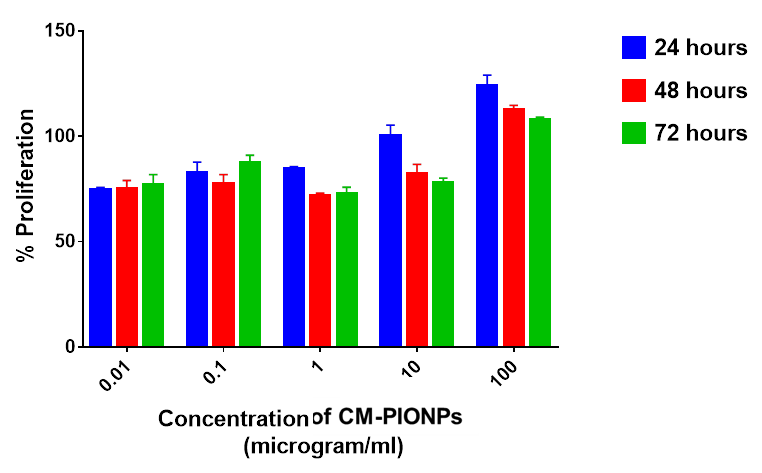
***

***Supplementary figure S10: Cell viability analysis of IC-21 macrophages treated with CM-PIONPs for 24, 48 and 72 hours***

******

***Supplementary figure S11: Cell viability analysis of cells treated with CM-PIONPs for 24, 48 and 72 hours***

******

***Supplementary figure S12: Cell viability analysis of (a) A549; (b) U-87MG and (c) B16F10 treated with DOX and DOX@CM-PIONPs for 24, 48 and 72 hours***


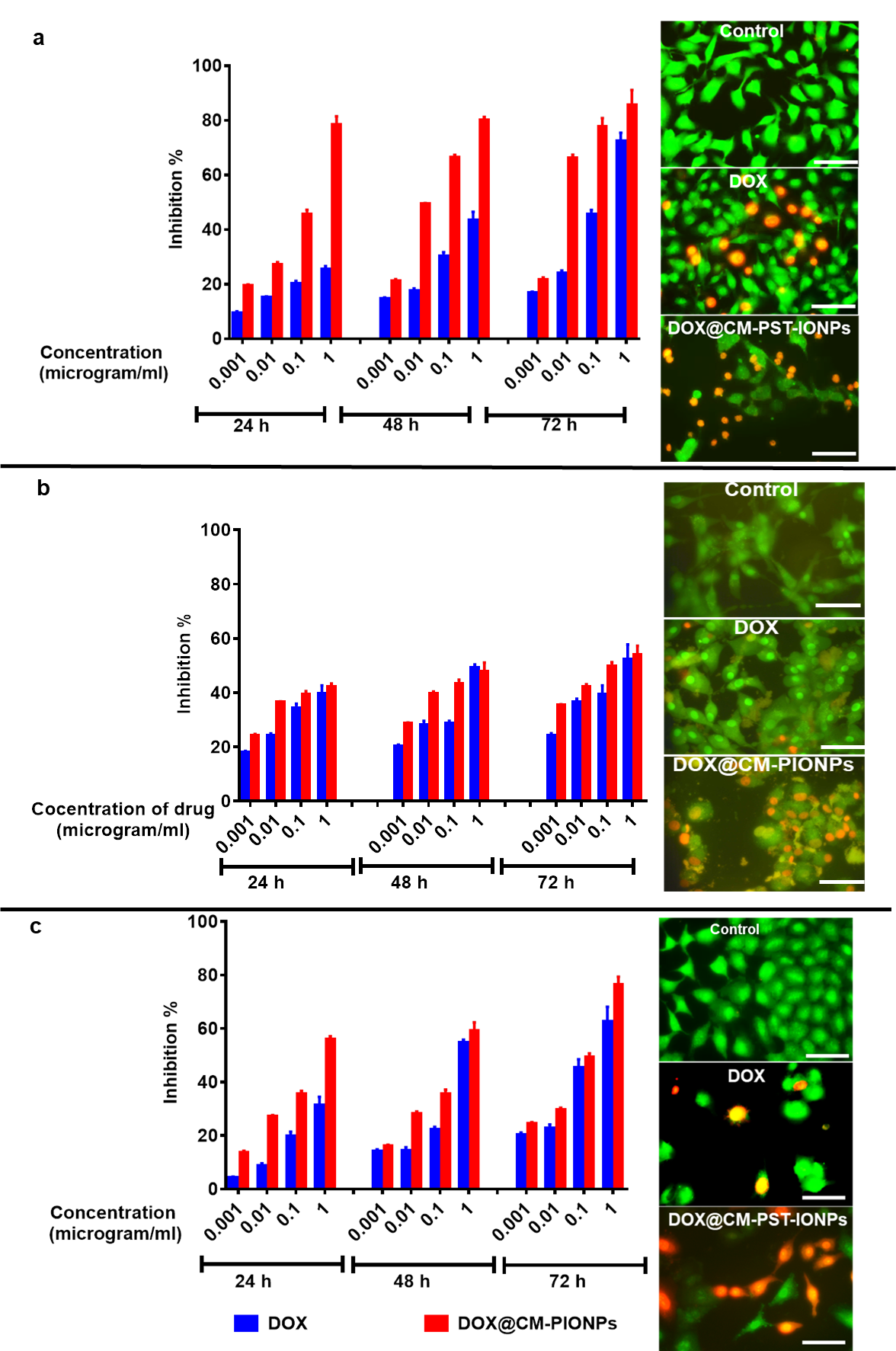


***Supplementary figure S13: Hoechst staining of (a) A549; (b) U-87MG and (c) B16F10 treated with CM-PIONPs and DOX@CM-PIONPs for 48 hours***


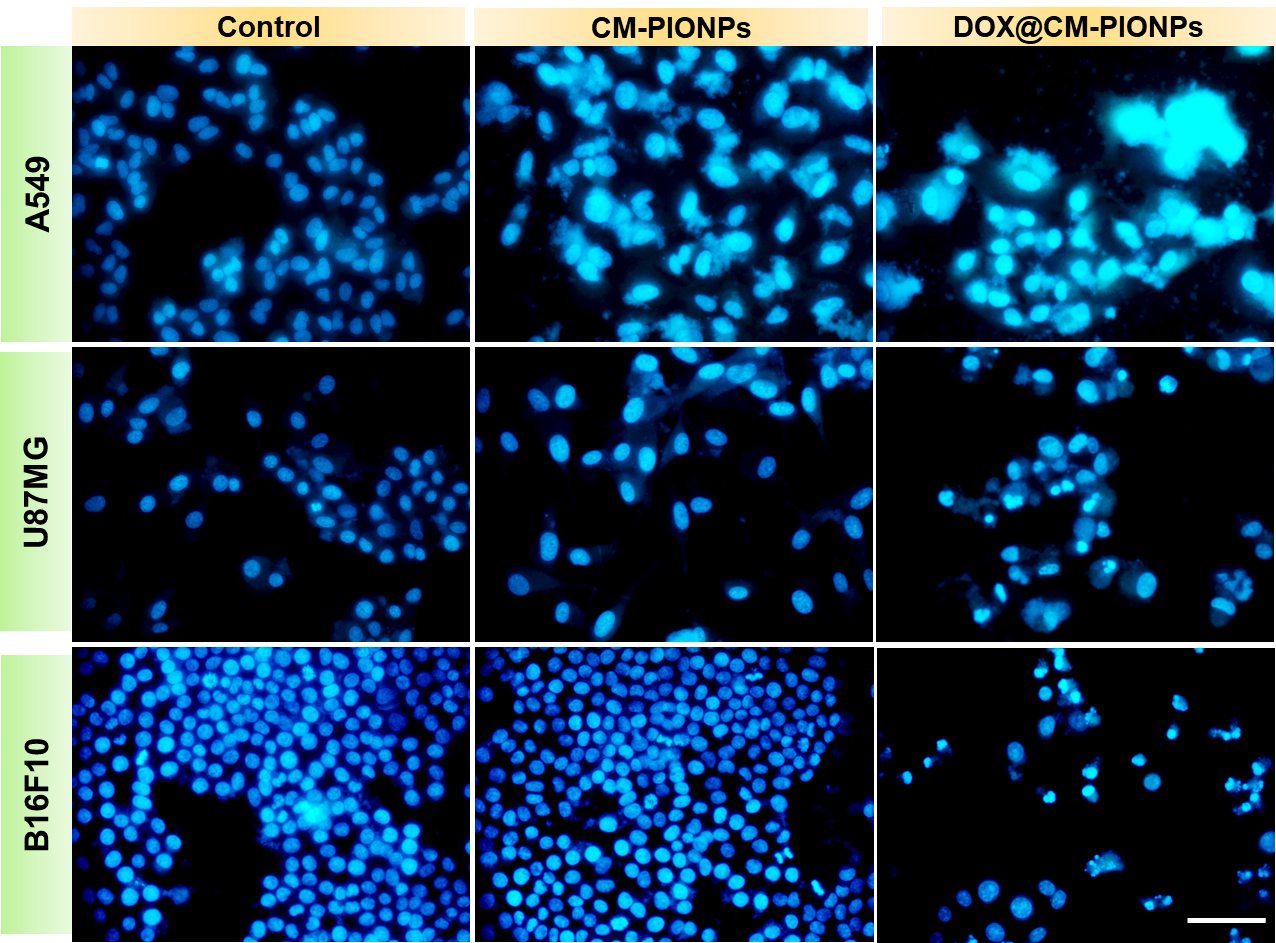


***Supplementary figure S14: Effect of DOX@CM-PIONPs in ROS generation: (a) qualitative analysis; (b) quantitative analysis***


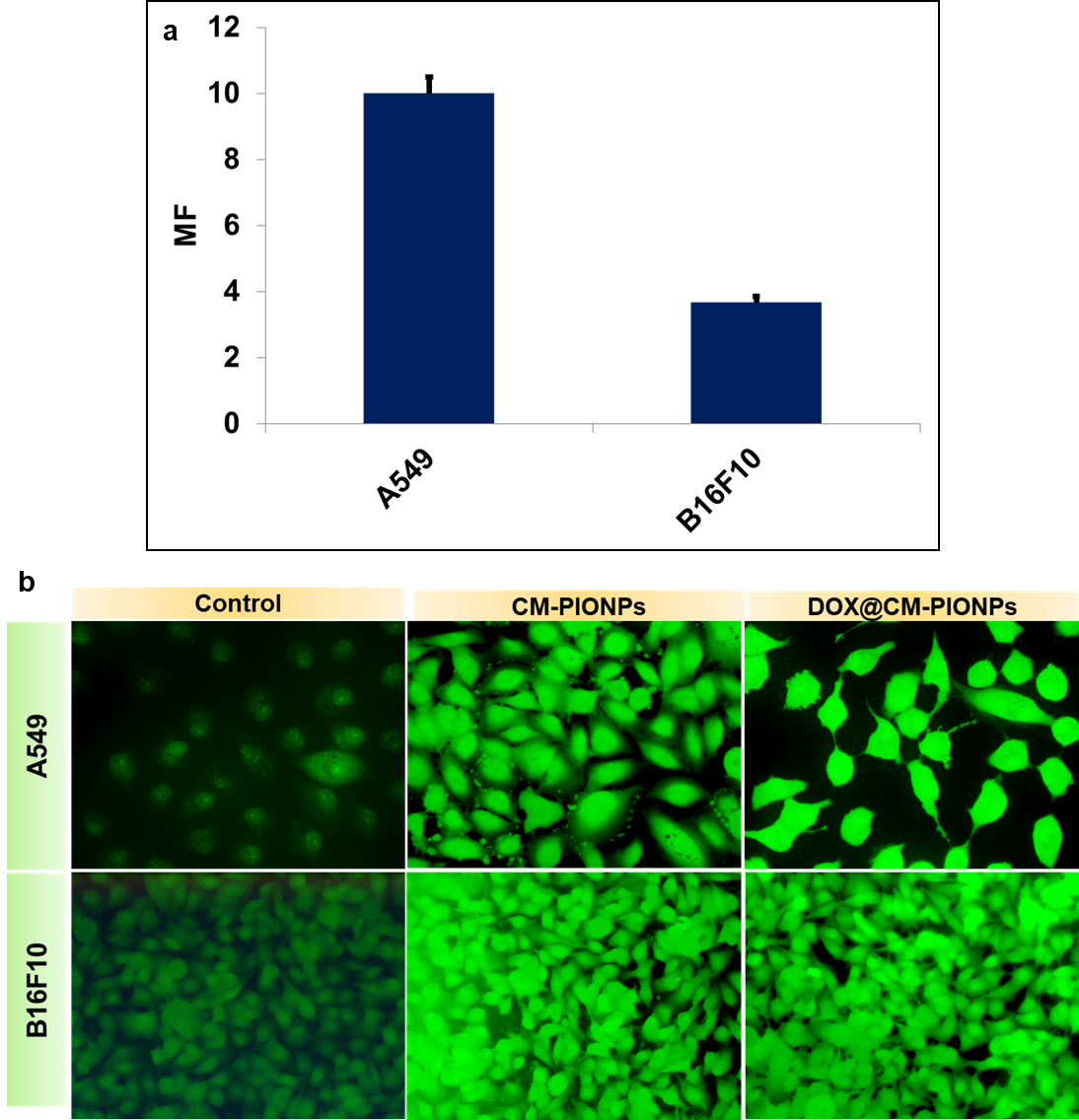


***Supplementary figure S15: SSC plot of flow cytometric analysis: A549 cells treated with DOX and DOX@CM-PIONPs for 3h were analyzed using FACS flow cytometer***


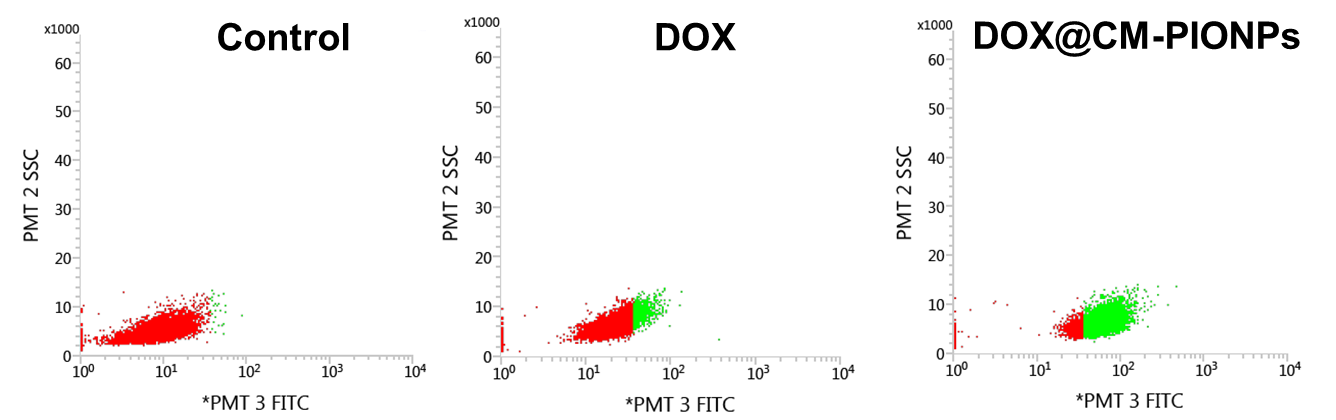


***Supplementary figure S16: Formation of glioma spheroids with increasing incubation time***

***Supplementary figure S17: Representative micrographs of spheroid formation***


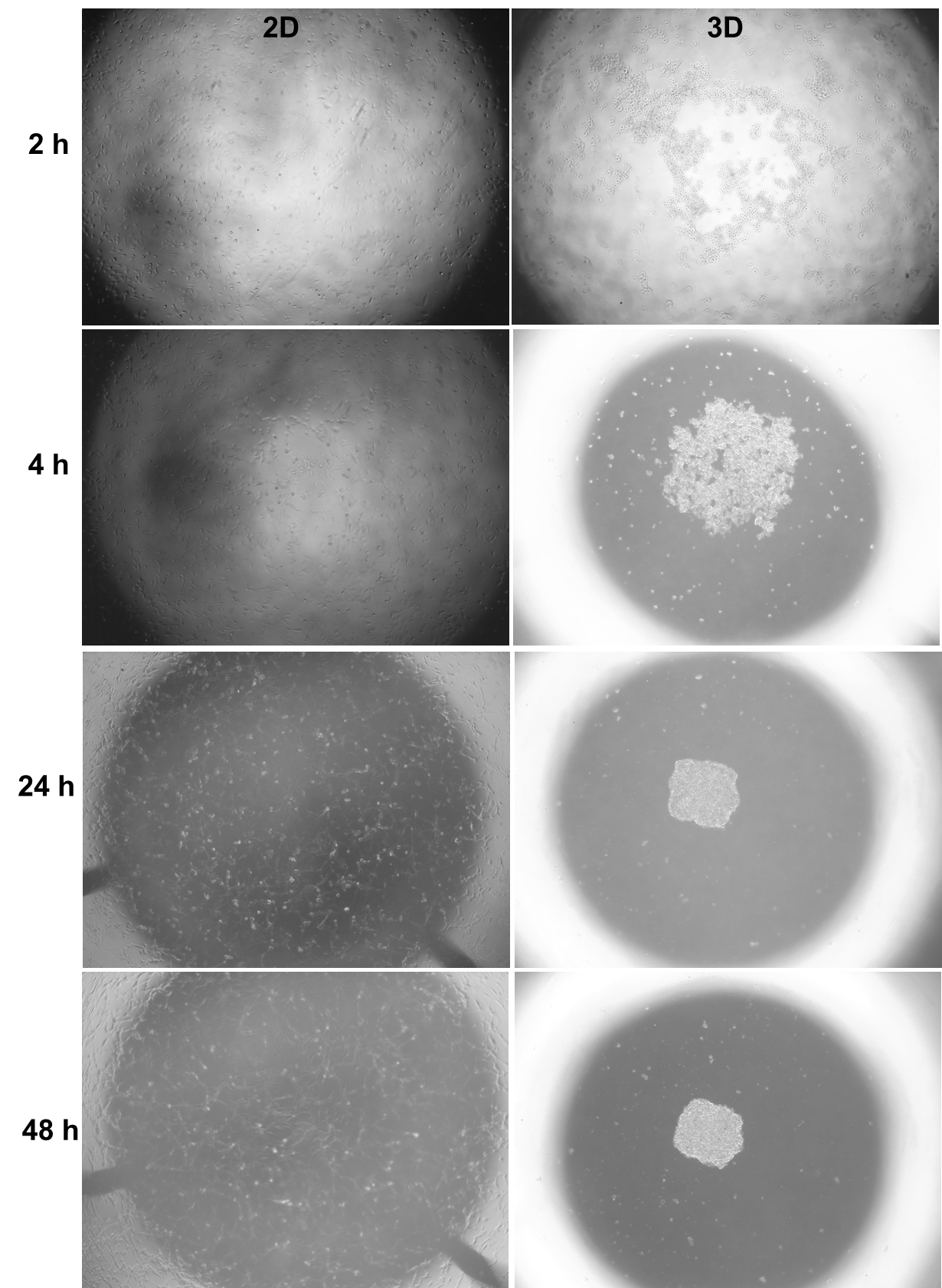


***Supplementary figure S18: Calcein AM staining; Phase contrast image of (a) U-87MG 2-D culture and (b) U-87MG 3-D culture; Calcein AM staining of (c) U-87MG 2-D culture and (d) U-87MG 3-D culture***


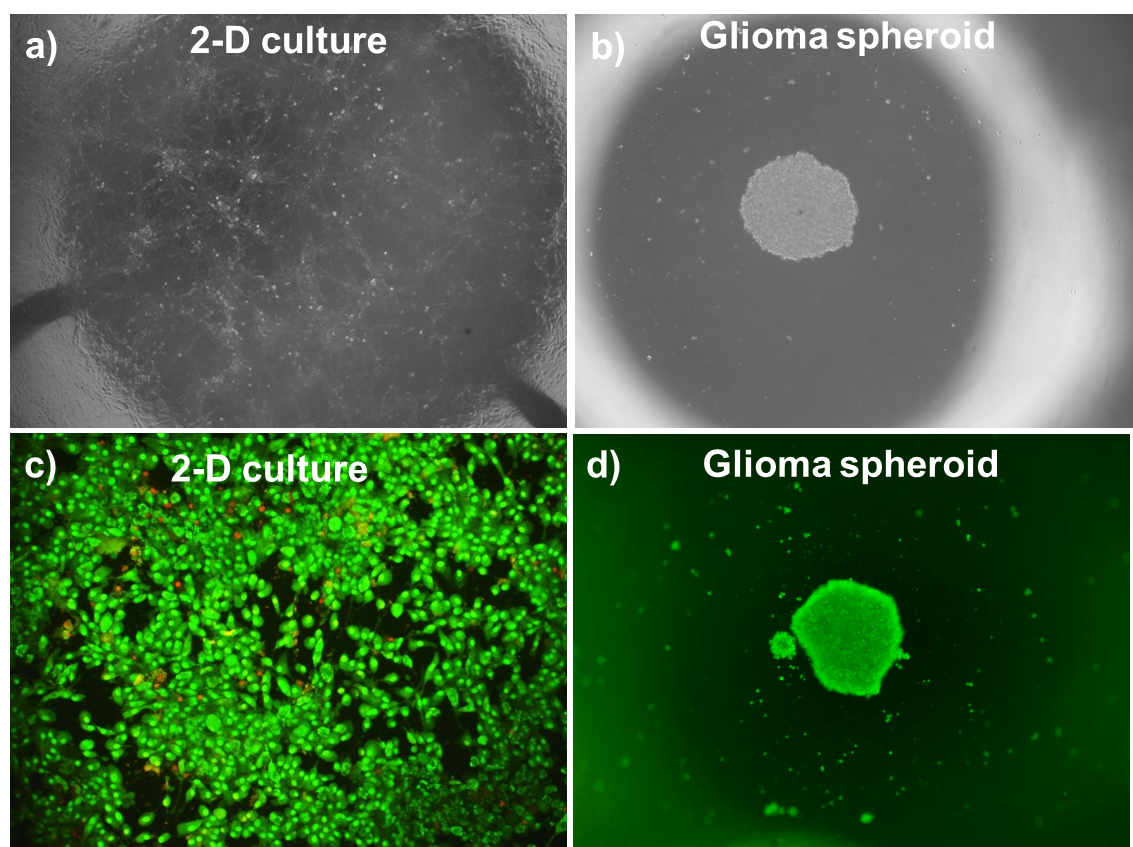


***Supplementary figure S19: Lipid peroxidation assay; (a) standard curve; (b) in vivo lipid peroxide levels after administration of DOX@CM-PIONPs***


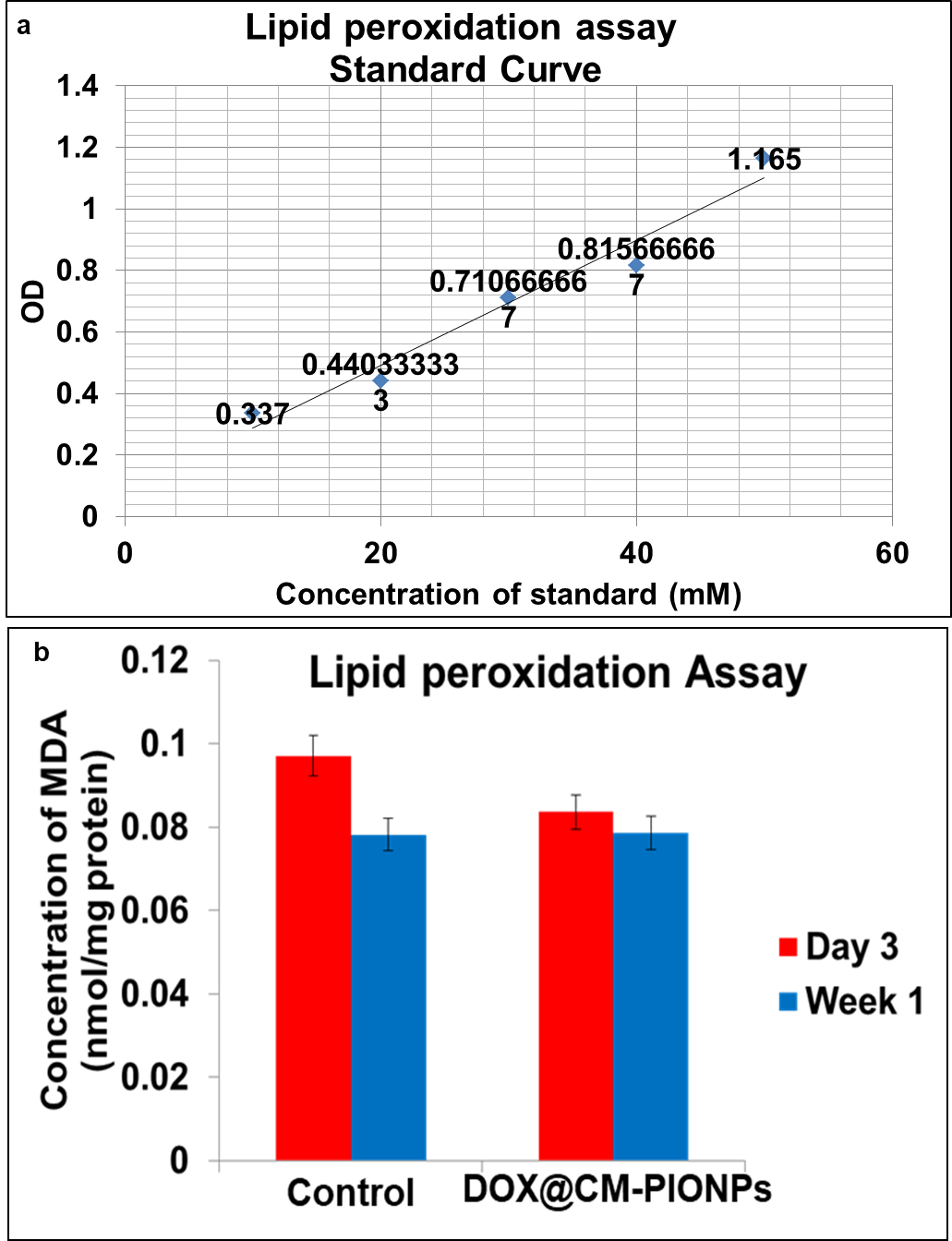


***Supplementary figure S20: Glutathione assay after administration of DOX@CM-PIONPs***


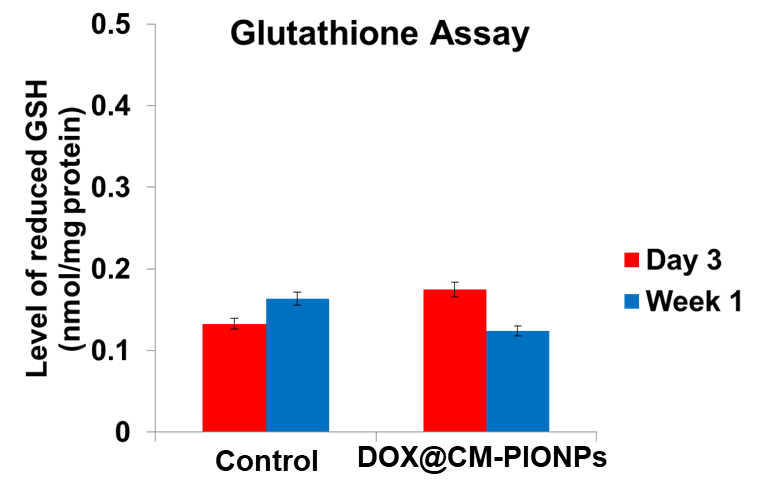


***Supplementary figure S21: Body weight change***

**Supplementary figure S22: Survival Analysis of DLA tumor-bearing mice intraperitonially injected with PBS, CM-PIONPs, DOX and DOX@CM-PIONPs (n=6)**

**References**

1. Aravind, S., et al., *TRAIL-based tumor sensitizing galactoxyloglucan, a novel entity for targeting apoptotic machinery.* The international journal of biochemistry & cell biology, 2015. **59**: p. 153-166.

2. Goyal, P., V. Kumar, and P. Sharma, *Carboxymethylation of tamarind kernel powder.* Carbohydrate polymers, 2007. **69**(2): p. 251-255.

3. Saraswathy, A., et al., *Synthesis and characterization of dextran stabilized superparamagnetic iron oxide nanoparticles for in vivo MR imaging of liver fibrosis.* Carbohydrate polymers, 2014. **101**: p. 760-768.
